# Balloon Angioplasty vs. Stenting for Symptomatic Intracranial Arterial Stenosis

**DOI:** 10.3389/fneur.2022.878179

**Published:** 2022-06-14

**Authors:** Yaxuan Sun, Xihua Li, Yongxia Ding, Bin Han, Jing Wang, Kun Meng, Yan Han

**Affiliations:** ^1^Department of Neurology, Shanxi Provincial People's Hospital, Taiyuan, China; ^2^The Fifth Clinical Medical College of Shanxi Medical University, Taiyuan, China; ^3^College of Nursing, Shanxi Medical University, Taiyuan, China

**Keywords:** intracranial arterial stenosis, balloon angioplasty, stenting, systematic review, meta-analysis

## Abstract

**Aims:**

We performed a meta-analysis to indirectly compare the treatment effectiveness of balloon angioplasty and stenting for patients with intracranial arterial stenosis.

**Methods:**

Literature searches were performed in well-known databases to identify eligible studies published before January 04, 2021. The incidence of restenosis, transient ischemic attack (TIA), stroke, death, and dissection after balloon angioplasty or stenting were pooled. An indirect comparison of balloon angioplasty vs. stenting was performed, and the ratios of incidence (RIs) with 95% confidence intervals (CIs) were calculated using the random-effects model.

**Results:**

120 studies that recruited 10,107 patients with intracranial arterial stenosis were included. The pooled incidence of restenosis after balloon angioplasty and stenting were 13% (95%CI: 8-17%) and 11% (95%CI: 9-13%), respectively, with no significant difference between them (RI: 1.18; 95%CI: 0.78–1.80; *P* = 0.435). Moreover, the pooled incidence of TIA after balloon angioplasty and stenting was 3% (95%CI: 0–6%) and 4% (95%CI: 3%-5%), and no significant difference was observed (RI: 0.75; 95%CI: 0.01–58.53; *P* = 0.897). The pooled incidence of stroke after balloon angioplasty and stenting was 7% (95%CI: 5–9%) and 8% (95%CI: 7–9%), respectively, and the difference between groups was found to be statistically insignificant (RI: 0.88; 95%CI: 0.64–1.20; *P* = 0.413). Additionally, the pooled incidence of death after balloon angioplasty and stenting was 2% (95%CI: 1–4%) and 2% (95%CI: 1–2%), with no significant difference between groups (RI: 1.00; 95%CI: 0.44–2.27; *P* = 1.000). Finally, the pooled incidence of dissection after balloon angioplasty and stenting was 13% (95%CI: 5–22%) and 3% (95%CI: 2–5%), respectively, and balloon angioplasty was associated with a higher risk of dissection than that with stenting for patients with intracranial arterial stenosis (RI: 4.33; 95%CI: 1.81–10.35; *P* = 0.001).

**Conclusion:**

This study found that the treatment effectiveness of balloon angioplasty and stenting were similar for patients with symptomatic intracranial arterial stenosis.

## Introduction

Intracranial stenosis of the major cerebral arteries is a significant risk factor for ischemic stroke (1–3), and it accounts for 10–54% of all ischemic strokes (4). The risk of stroke mortality shows regional variation and is disproportionately high in Asian countries, which might contribute to the high prevalence of intracranial stenosis in Asia (5). In China, extracranial and intracranial artery stenosis was common for symptomatic patients, and the most common location for intracranial stenosis was the internal carotid artery (6, 7). The high prevalence of intracranial artery stenosis among Asian and African-American populations could be explained by genetic susceptibility, environmental factors, and high rate of hypertension, diabetes mellitus, and hyperlipidemia (8–10). Strokes caused by intracranial artery stenosis are responsible for great economic and familial burdens, especially in low or middle-income countries (11).

Current treatment strategies for intracranial artery stenosis include medical, surgical, and endovascular therapies. Endovascular therapy, including balloon angioplasty and stenting, is a minimally invasive approach and has acceptable periprocedural complication rates (12–14). Studies have already illustrated the prognosis outcomes after balloon angioplasty and stenting for patients with symptomatic intracranial artery stenosis (15, 16), but the difference between balloon angioplasty and stenting regarding the risk of restenosis, transient ischemic attack (TIA), stroke, death, and dissection were not illustrated. Therefore, we performed an indirect comparison meta-analysis to compare the effects of balloon angioplasty with that of stenting for symptomatic intracranial artery stenosis.

## Materials and Methods

### Search Strategy and Selection Criteria

This study was performed and reported according to the Preferred Reporting Items for Systematic Reviews and Meta-Analysis guidelines (17). Studies assessing the effects of balloon angioplasty or stenting for symptomatic intracranial artery stenosis were eligible for our study, and no restrictions were placed on publication language and status. The databases of PubMed, EmBase, and Cochrane library were systematically searched from their inception until January 04, 2021 for eligible studies. The following search terms were used: intracranial artery stenosis, balloon angioplasty, and stent. The details of the search strategy used in PubMed are shown in Supplementary Material. The reference lists of relevant reviews and original articles were also reviewed manually for additional eligible studies.

The literature search and study selection was performed independently by two reviewers, and conflicts between reviewers were settled by mutual discussion until a consensus was reached. Studies were included if they met the following inclusion criteria: (1) patients with symptomatic intracranial artery stenosis; (2) interventions including balloon angioplasty or stenting; and (3) outcomes including restenosis, TIA, stroke, death, and dissection. No restrictions were placed on study design.

### Data Collection and Quality Assessment

The following information was independently extracted from selected papers by two reviewers: first author, publication year, country, study design, sample size, mean participant age, proportion of male participants, preprocedural stenosis, lesion location, history of hypertension, diabetes mellitus, and smoking, intervention, follow-up, and the incidences of restenosis, TIA, stroke, death, and dissection. Then these two reviewers independently assessed the quality of individual study using the Newcastle-Ottawa Scale (NOS) for selection process, comparability, and outcome, and the starring system for each study ranged from 0 to 9 (18). Studies with 6–7 stars were regarded to have moderate quality, and those with 4–5 stars were considered to be of low quality. Any disagreement between reviewers was resolved by an additional reviewer referring to the original article.

### Statistical Analysis

The incidences, after balloon angioplasty or stenting, of restenosis, TIA, stroke, death, and dissection were calculated based on event occurrence and sample size. Pooled incidence was calculated using the random-effects model (19, 20). The variability of pooled conclusions was calculated by the sequential removal of single studies (21). Subgroup analyses were also performed based on pre-defined variables, including publication year, country, study design, mean participant age, proportion of male participants, preprocedural stenosis, lesion location, hypertension, diabetes mellitus, smoking, and study quality. An indirect comparison was conducted, and ratios of incidence (RIs) with 95% confidence intervals (CIs) were calculated (22). Heterogeneity of included studies was assessed using I^2^ and Q statistics, and significant heterogeneity was defined as I^2^ > 50.0% or *P* < 0.10 (23, 24). Publication bias for the incidence of restenosis, TIA, stroke, death, and dissection were assessed using funnel plots and Egger and Begg tests (25, 26). The inspection level for pooled outcomes were two-sided, and P < 0.05 was considered statistically significant. All analyses in this study were performed using the STATA software package (version 10.0; Stata Corporation, College Station, TX, USA).

## Results

### Search of the Published Literature

**Figure 1:**
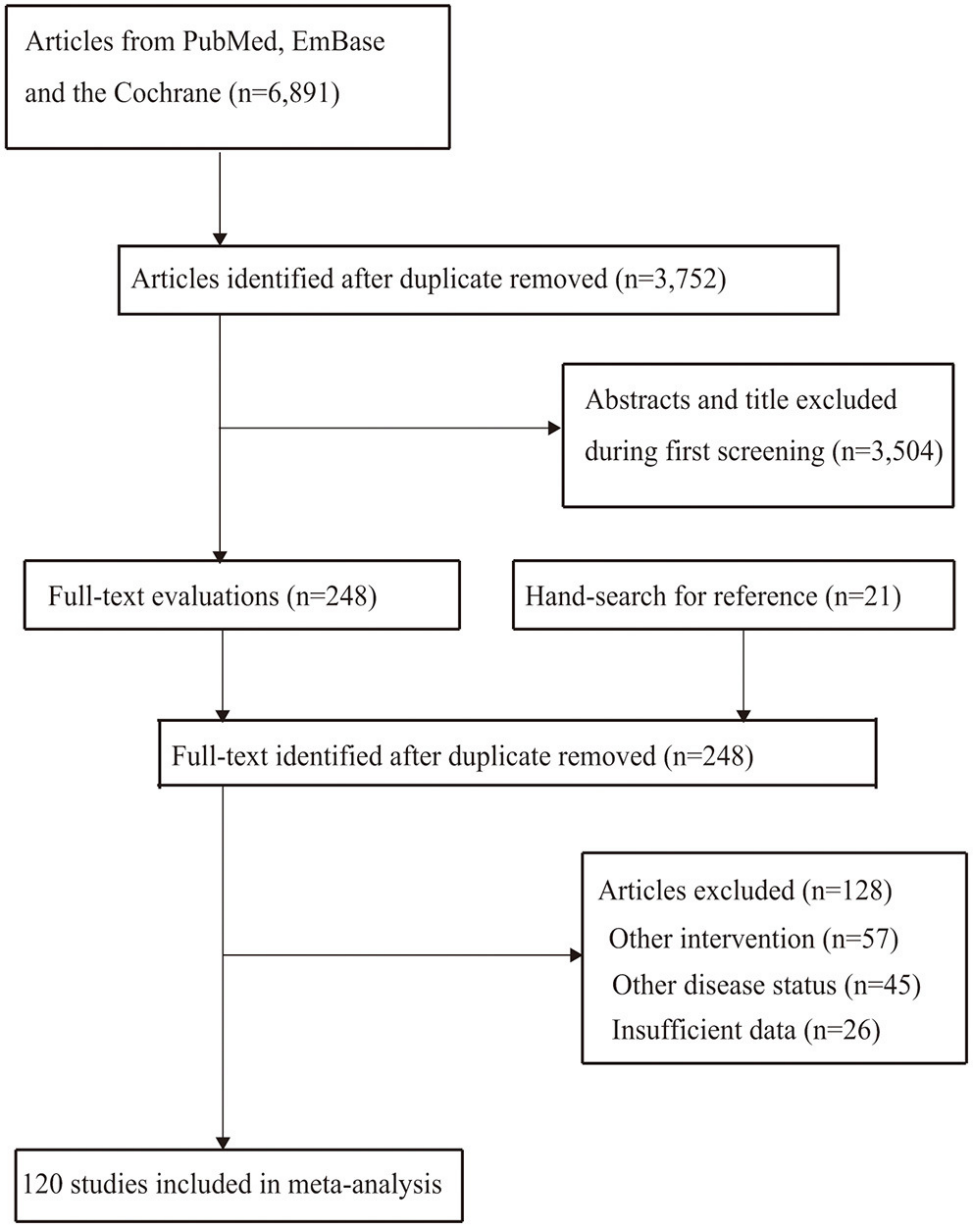
PRISMA flowchart for process of literature search and study selection.

A total of 6,891 articles were identified from the electronic databases, and 3,752 articles were retained after removal of duplicates. Thereafter, 3,504 articles were excluded because these studies reported on irrelevant topics. The remaining 248 studies were retrieved for further full-text evaluations, and 128 studies were removed owing to following reasons: other interventions (*n* = 57); other disease status (*n* = 45); and insufficient data (*n* = 26). The remaining 120 studies were selected for the final meta-analysis, and the details regarding this selection process are shown in Figure 1.

### Study Characteristics

The characteristics of included studies are summarized in Supplementary Material and Table 1. Overall, a total of 10,107 patients with intracranial arterial stenosis were recruited. The effects of balloon angioplasty and stenting were reported by 26 and 100 studies, respectively. The sample sizes for balloon angioplasty and stenting were 1,029 and 9,078 patients, respectively. Twenty-nine studies were prospective, while the remaining 91 studies were retrospective. The distribution of the NOS scale scores for the studies is shown in Table 1.

**Table 1:** Summary of characteristics of identified individuals.

**Variables**	**Groups**	**BA**	**Stent**
Number of studies	–	26	100
Country	Western	13	37
	Eastern	13	63
Study design	Prospective	1	29
	Retrospective	25	71
Sample size	–	1,029	9,078
Mean age (years)	≥65.0	4	26
	<65.0	17	70
	NA	5	4
Male (%)	≥80.0	4	25
	<80.0	20	72
	NA	2	3
Preprocedural stenosis (%)	≥80.0	13	26
	<80.0	13	74
Lesion location	≥3 sites	13	64
	<3 sites	13	36
Hypertension (%)	≥80.0	3	18
	<80.0	6	39
	NA	17	43
DM (%)	≥40.0	3	17
	<40.0	6	39
	NA	17	44
Smoking (%)	≥40.0	1	20
	<40.0	5	31
	NA	20	49
Study quality	7	3	14
	6	5	27
	5	14	43
	4	4	16

*BA, balloon angioplasty; DM, diabetes mellitus*.

### Restenosis

**Figure 2:**
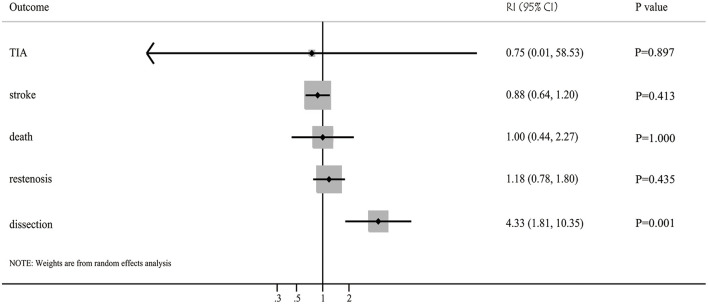
Balloon angioplasty vs. stenting with regards to the risk of restenosis, TIA, stroke, death, and dissection for patients with symptomatic intracranial arterial stenosis.

The incidence of restenosis after balloon angioplasty or stenting was reported in 19 and 50 studies, respectively (Supplementary Material). The pooled incidence of restenosis after balloon angioplasty and stenting was 13% (95%CI: 8–17%) and 11% (95%CI: 9–13%), respectively. There was no significant difference between balloon angioplasty and stenting for the risk of restenosis (RI: 1.18; 95%CI: 0.78–1.80; *P* = 0.435; Figure 2). Sensitivity analyses indicated that the pooled incidence of restenosis after balloon angioplasty and stenting ranged from 10.4 to 13.8%, and 10.0 to 10.9%, respectively (Supplementary Material). Subgroup analysis showed that the differences in incidences of restenosis after balloon angioplasty and stenting were not significant for any subgroup (Table 2). There was significant publication bias for the incidence of restenosis after balloon angioplasty and stenting (Supplementary Material).

**Table 2:** Outcomes for balloon angioplasty vs. stenting according to study or patients' characteristics.

**Outcomes**	**Variables**	**Groups**	**BA**	**Stent**	**RI (95%CI)**	***P* value**
Restenosis	Publication year	2005 or before	0.11 (0.06–0.17)	0.10 (0.04–0.16)	1.10 (0.46–2.62)	0.829
		After 2005	0.14 (0.07–0.21)	0.11 (0.09–0.13)	1.27 (0.71–2.27)	0.415
	Country	Western	0.10 (0.06–0.14)	0.12 (0.08–0.16)	0.83 (0.48–1.44)	0.514
		Eastern	0.16 (0.08–0.23)	0.10 (0.08–0.12)	1.60 (0.91–2.82)	0.103
	Study design	Prospective	–	0.14 (0.10–0.18)	–	–
		Retrospective	0.13 (0.08–0.17)	0.10 (0.08–0.12)	1.30 (0.85–1.99)	0.230
	Mean age (years)	≥ 65.0	0.21 (0.11–0.30)	0.12 (0.07–0.16)	1.75 (0.91–3.35)	0.092
		<65.0	0.13 (0.07–0.18)	0.10 (0.08–0.13)	1.30 (0.78–2.21)	0.333
	Male participants (%)	≥ 80.0	0.16 (0.00–0.41)	0.08 (0.05–0.12)	2.00 (0.03–131.04)	0.745
		<80.0	0.14 (0.09–0.19)	0.12 (0.09–0.14)	1.17 (0.76–1.80)	0.486
	Preprocedural stenosis (%)	≥ 80.0	0.15 (0.07–0.22)	0.11 (0.08–0.15)	1.36 (0.71–2.62)	0.352
		<80.0	0.13 (0.05–0.21)	0.10 (0.08–0.13)	1.30 (0.61–2.77)	0.497
	Lesion location	≥ 3 sites	0.09 (0.04–0.13)	0.10 (0.08–0.13)	0.90 (0.48–1.70)	0.746
		<3 sites	0.17 (0.09–0.24)	0.11 (0.08–0.14)	1.55 (0.88–2.72)	0.131
	Hypertension (%)	≥ 80.0	0.03 (0.00–0.06)	0.12 (0.07–0.17)	0.25 (0.03–2.03)	0.195
		<80.0	0.19 (0.08–0.29)	0.11 (0.08–0.15)	1.73 (0.84–3.54)	0.135
	DM (%)	≥ 40.0	0.03 (0.00–0.06)	0.14 (0.08–0.21)	0.21 (0.03–1.76)	0.151
		<40.0	0.15 (0.06–0.25)	0.10 (0.07–0.13)	1.50 (0.69–3.27)	0.307
	Smoking (%)	≥ 40.0	–	0.15 (0.08–0.21)	–	–
		<40.0	0.11 (0.00–0.22)	0.11 (0.08–0.15)	1.00 (0.07–15.11)	1.000
	Study quality	Moderate	0.15 (0.05–0.26)	0.15 (0.10–0.19)	1.00 (0.41–2.42)	1.000
		Low	0.12 (0.07–0.17)	0.08 (0.06–0.10)	1.50 (0.90–2.50)	0.121
TIA	Publication year	2005 or before	0.03 (0.01–0.07)	0.09 (0.00–0.20)	0.33 (0.02–5.60)	0.445
		After 2005	0.04 (0.00–0.06)	0.04 (0.03–0.05)	1.00 (0.13–7.87)	1.000
	Country	Western	0.02 (0.00–0.04)	0.04 (0.02–0.07)	0.50 (0.07–3.51)	0.486
		Eastern	0.09 (0.02–0.15)	0.03 (0.02–0.04)	3.00 (1.03–8.71)	0.043
	Study design	Prospective	–	0.03 (0.02–0.04)	–	–
		Retrospective	0.03 (0.00–0.06)	0.04 (0.03–0.06)	0.75 (0.09–5.98)	0.786
	Mean age (years)	≥ 65.0	–	0.06 (0.02–0.10)	–	–
		<65.0	0.05 (0.00–0.10)	0.03 (0.03–0.04)	1.67 (0.16–16.87)	0.665
	Male participants (%)	≥ 80.0	–	0.03 (0.01–0.05)	–	–
		<80.0	0.05 (0.00–0.10)	0.04 (0.03–0.05)	1.25 (0.12–12.68)	0.850
	Preprocedural stenosis (%)	≥80.0	0.09 (0.02–0.15)	0.04 (0.03–0.05)	2.25 (0.80–6.36)	0.126
		<80.0	0.01 (0.00–0.04)	0.04 (0.02–0.06)	0.25 (0.04–1.61)	0.145
	Lesion location	≥3 sites	0.02 (0.00–0.04)	0.04 (0.03–0.05)	0.50 (0.08–3.22)	0.466
		<3 sites	0.09 (0.02–0.15)	0.03 (0.02–0.05)	3.00 (0.99–9.07)	0.052
	Hypertension (%)	≥80.0	0.01 (0.00–0.04)	0.04 (0.02–0.06)	0.25 (0.04–1.71)	0.158
		<80.0	0.08 (0.02–0.15)	0.04 (0.02–0.06)	2.00 (0.63–6.30)	0.236
	DM (%)	≥40.0	0.01 (0.00–0.04)	0.18 (0.00–0.36)	0.06 (0.00–1.79)	0.103
		<40.0	0.08 (0.02–0.15)	0.04 (0.02–0.05)	2.00 (0.66–6.05)	0.220
	Smoking (%)	≥40.0	–	0.04 (0.02–0.06)	–	–
		<40.0	0.01 (0.00–0.04)	0.04 (0.02–0.06)	0.25 (0.04–1.71)	0.158
	Study quality	Moderate	0.01 (0.00–0.04)	0.03 (0.02–0.04)	0.33 (0.05–2.18)	0.251
		Low	0.05 (0.01–0.09)	0.05 (0.03–0.06)	1.00 (0.32–3.16)	1.000
Stroke	Publication year	2005 or before	0.10 (0.05–0.15)	0.06 (0.01–0.11)	1.67 (0.45–6.23)	0.448
		After 2005	0.07 (0.05–0.09)	0.08 (0.07–0.10)	0.88 (0.62–1.23)	0.446
	Country	Western	0.09 (0.06–0.12)	0.12 (0.09–0.14)	0.75 (0.50–1.13)	0.170
		Eastern	0.06 (0.04–0.09)	0.07 (0.06–0.08)	0.86 (0.56–1.32)	0.483
	Study design	Prospective	–	0.09 (0.07–0.11)	–	–
		Retrospective	0.07 (0.05–0.09)	0.08 (0.07–0.09)	0.88 (0.64–1.20)	0.413
	Mean age (years)	≥65.0	0.06 (0.00–0.12)	0.11 (0.08–0.13)	0.55 (0.11–2.72)	0.460
		<65.0	0.07 (0.05–0.10)	0.08 (0.07–0.09)	0.88 (0.61–1.27)	0.478
	Male participants (%)	≥80.0	0.12 (0.04–0.20)	0.06 (0.04–0.08)	2.00 (0.83–4.80)	0.121
		<80.0	0.07 (0.05–0.10)	0.09 (0.08–0.10)	0.78 (0.54–1.12)	0.176
	Preprocedural stenosis (%)	≥80.0	0.08 (0.04–0.12)	0.08 (0.06–0.10)	1.00 (0.55–1.83)	1.000
		<80.0	0.07 (0.04–0.10)	0.08 (0.07–0.10)	0.88 (0.54–1.43)	0.594
	Lesion location	≥3 sites	0.07 (0.04–0.10)	0.08 (0.07–0.10)	0.88 (0.54–1.43)	0.594
		<3 sites	0.08 (0.05–0.12)	0.08 (0.07–0.10)	1.00 (0.62–1.60)	1.000
	Hypertension (%)	≥80.0	0.08 (0.04–0.11)	0.12 (0.08–0.15)	0.67 (0.37–1.21)	0.182
		<80.0	0.08 (0.04–0.13)	0.07 (0.06–0.08)	1.14 (0.62–2.10)	0.666
	DM (%)	≥40.0	0.08 (0.04–0.12)	0.11 (0.06–0.15)	0.73 (0.36–1.49)	0.383
		<40.0	0.07 (0.04–0.11)	0.08 (0.07–0.09)	0.88 (0.52–1.47)	0.616
	Smoking (%)	≥40.0	–	0.07 (0.05–0.08)	–	–
		<40.0	0.08 (0.05–0.11)	0.10 (0.08–0.12)	0.80 (0.51–1.25)	0.324
	Study quality	Moderate	0.08 (0.05–0.10)	0.08 (0.06–0.09)	1.00 (0.67–1.49)	1.000
		Low	0.08 (0.05–0.11)	0.09 (0.07–0.11)	0.89 (0.56–1.40)	0.611
Death	Publication year	2005 or before	0.04 (0.02–0.07)	0.05 (0.01–0.08)	0.80 (0.24–2.69)	0.719
		After 2005	0.02 (0.00–0.03)	0.02 (0.01–0.02)	1.00 (0.33–3.00)	1.000
	Country	Western	0.02 (0.02–0.05)	0.03 (0.02–0.04)	0.67 (0.33–1.34)	0.253
		Eastern	0.04 (0.01–0.06)	0.01 (0.01–0.02)	4.00 (1.29–12.42)	0.016
	Study design	Prospective	–	0.02 (0.02–0.03)	–	–
		Retrospective	0.02 (0.01–0.04)	0.02 (0.01–0.02)	1.00 (0.44–2.30)	1.000
	Mean age (years)	≥65.0	0.03 (0.00–0.07)	0.02 (0.01–0.04)	1.50 (0.16–14.01)	0.722
		<65.0	0.02 (0.00–0.04)	0.02 (0.01–0.03)	1.00 (0.15–6.85)	1.000
	Male participants (%)	≥80.0	0.09 (0.00–0.18)	0.01 (0.01–0.02)	9.00 (0.61–132.24)	0.109
		<80.0	0.02 (0.01–0.04)	0.02 (0.02–0.03)	1.00 (0.46–2.17)	1.000
	Preprocedural stenosis (%)	≥80.0	0.03 (0.00–0.06)	0.02 (0.01–0.02)	1.76 (0.22–14.32)	0.595
		<80.0	0.03 (0.01–0.06)	0.02 (0.02–0.03)	1.50 (0.57–3.92)	0.408
	Lesion location	≥3 sites	0.04 (0.01–0.06)	0.02 (0.01–0.03)	2.00 (0.70–5.72)	0.196
		<3 sites	0.02 (0.00–0.04)	0.02 (0.01–0.02)	1.11 (0.17–7.40)	0.913
	Hypertension (%)	≥80.0	0.03 (0.00–0.06)	0.03 (0.02–0.04)	1.00 (0.13–7.97)	1.000
		<80.0	0.02 (0.00–0.05)	0.01 (0.01–0.02)	2.00 (0.27–14.91)	0.499
	DM (%)	≥40.0	0.03 (0.00–0.07)	0.02 (0.01–0.04)	1.50 (0.16–14.01)	0.722
		<40.0	0.02 (0.00–0.05)	0.02 (0.01–0.02)	1.00 (0.13–7.46)	1.000
	Smoking (%)	≥40.0	–	0.01 (0.01–0.02)	–	–
		<40.0	0.02 (0.00–0.03)	0.02 (0.01–0.03)	1.00 (0.17–5.97)	1.000
	Study quality	Moderate	0.01 (0.00–0.03)	0.02 (0.01–0.02)	0.50 (0.09–2.91)	0.440
		Low	0.04 (0.02–0.06)	0.02 (0.02–0.03)	2.00 (1.04–3.83)	0.036
Dissection	Publication year	2005 or before	0.19 (0.07–0.31)	–	–	–
		After 2005	0.03 (0.00–0.06)	0.03 (0.02–0.05)	1.00 (0.17–5.82)	1.000
	Country	Western	0.15 (0.02–0.28)	0.05 (0.02–0.07)	3.00 (0.70–12.93)	0.140
		Eastern	0.10 (0.00–0.23)	0.03 (0.01–0.05)	3.33 (0.42–26.59)	0.256
	Study design	Prospective	–	0.02 (0.00–0.05)	–	–
		Retrospective	0.13 (0.05–0.22)	0.04 (0.02–0.06)	3.25 (1.29–8.17)	0.012
	Mean age (years)	≥65.0	0.10 (0.00–0.23)	0.05 (0.01–0.08)	2.00 (0.23–17.67)	0.533
		<65.0	0.05 (0.02–0.12)	0.04 (0.02–0.06)	1.25 (0.44–3.58)	0.677
	Male participants (%)	≥80.0	0.18 (0.00–0.36)	0.06 (0.01–0.10)	3.00 (0.26–34.03)	0.375
		<80.0	0.07 (0.00–0.13)	0.03 (0.01–0.05)	2.33 (0.38–14.36)	0.361
	Preprocedural stenosis (%)	≥80.0	0.07 (0.00–0.15)	0.07 (0.00–0.16)	1.00 (0.09–11.34)	1.000
		<80.0	0.10 (0.00–0.19)	0.03 (0.01–0.04)	3.33 (0.48–23.34)	0.225
	Lesion location	≥3 sites	0.15 (0.00–0.35)	0.02 (0.01–0.03)	7.50 (0.84–67.29)	0.072
		<3 sites	0.11 (0.01–0.21)	0.06 (0.03–0.10)	1.83 (0.36–9.42)	0.468
	Hypertension (%)	≥80.0	0.04 (0.00–0.11)	0.07 (0.00–0.14)	0.57 (0.06–5.55)	0.629
		<80.0	–	0.06 (0.00–0.17)	–	–
	DM (%)	≥40.0	–	0.08 (0.00–0.15)	–	–
		<40.0	0.04 (0.00–0.11)	0.06 (0.00–0.17)	0.67 (0.06–6.95)	0.735
	Smoking (%)	≥40.0	–	0.13 (0.05–0.20)	–	–
		<40.0	–	0.05 (0.00–0.10)	–	–
	Study quality	Moderate	0.40 (0.15–0.65)	0.03 (0.00–0.06)	13.33 (3.15–56.43)	<0.001
		Low	0.11 (0.03–0.19)	0.04 (0.01–0.06)	2.75 (0.76–9.95)	0.123

*BA, balloon angioplasty; DM, diabetes mellitus; RI, ratio of incidence; CI, confidence intervals*.

### TIA

The incidence of TIA after balloon angioplasty and stenting was reported in 4 and 33 studies, respectively (Supplementary Material). The pooled incidence of TIA after balloon angioplasty and stenting was 3% (95%CI: 0–6%) and 4% (95%CI: 3–5%), respectively, and no significant difference between balloon angioplasty and stenting for the risk of TIA was observed (RI: 0.75; 95%CI: 0.01–58.53; *P* = 0.897; Figure 2). Sensitivity analyses indicated the incidence of pooled TIA after balloon angioplasty ranged from 2.0 to 5.0%, while after stenting ranged from 3.4 to 3.9% (Supplementary Material). Subgroup analysis showed that balloon angioplasty was associated with higher risk of TIA than that seen with stenting in Eastern countries (RI: 3.00; 95%CI: 1.03–8.71; *P* = 0.043; Table 2). There was no significant publication bias for TIA after balloon angioplasty, while significant publication bias for TIA after stenting was observed (Supplementary Material).

### Stroke

The incidence of stroke after balloon angioplasty and stenting was reported in 19 and 84 studies, respectively (Supplementary Material). The pooled incidence of stroke after balloon angioplasty and stenting was 7% (95%CI: 5–9%) and 8% (95%CI: 7–9%), respectively, and no significant difference was found between balloon angioplasty and stenting for the risk of stroke (RI: 0.88; 95%CI: 0.64–1.20; *P* = 0.413; Figure 2). Sensitivity analyses indicated that the pooled incidence of stroke after balloon angioplasty ranged from 7.0 to 7.8%, while after stenting it ranged from 7.9 to 8.5% (Supplementary Material). Subgroup analysis indicated no significant difference between balloon angioplasty and stenting for the risk of stroke in any subgroup (Table 2). There was significant publication bias for the pooled incidence of stroke after balloon angioplasty and stenting (Supplementary Material).

### Death

The incidence of death after balloon angioplasty and stenting was reported in 11 and 53 studies, respectively (Supplemental Material). The pooled incidence of death after balloon angioplasty and stenting was 2% (95%CI: 1–4%) and 2% (95%CI: 1–2%), respectively, and no significant difference between groups was found for the risk of death (RI: 1.00; 95%CI: 0.44–2.27; *P* = 1.000; Figure 2). Sensitivity analyses indicated the pooled incidence of death after balloon angioplasty and stenting ranged from 2.3 to 3.6%, and 1.8 to 2.0%, respectively (Supplementary Material). Subgroup analyses indicated that balloon angioplasty was associated with a higher risk of death than stenting was in studies conducted in Eastern countries (RI: 4.00; 95%CI: 1.29–12.42; *P* = 0.016), and those deemed to be of low quality (RI: 2.00; 95%CI: 1.04–3.83; *P* = 0.036) (Table 2). There was significant publication bias for the pooled incidence of death after balloon angioplasty and stenting (Supplementary Material).

### Dissection

The incidence of dissection after balloon angioplasty and stenting was reported in 8 and 12 studies, respectively (Supplementary Material). The pooled incidence of dissection after balloon angioplasty and stenting was 13% (95%CI: 5–22%) and 3% (95%CI: 2–5%), respectively, and balloon angioplasty was associated with an increased risk of dissection compared to that with stenting (RI: 4.33; 95%CI: 1.81–10.35; *P* = 0.001; Figure 2). Sensitivity analyses revealed that the pooled incidence of dissection after balloon angioplasty ranged from 7.7 to 15.9%, while after stenting, it ranged from 2.2 to 3.8% (Supplementary Material). Subgroup analyses showed that balloon angioplasty was associated with an increased risk of dissection when compared with that of stenting in studies with a retrospective design (RI: 3.25; 95%CI: 1.29–8.17; *P* = 0.012) and those deemed to be of moderate quality (RI: 13.33; 95%CI: 3.15–56.43; *P* < 0.001) (Table 2). The publication bias for pooled incidence of dissection after balloon angioplasty was not found to be statistically significant, while significant publication bias for dissection after stenting was seen (Supplementary Material).

## Discussion

This study aimed to compare the effects of balloon angioplasty with that of stenting for patients with symptomatic intracranial arterial stenosis. A total of 120 studies involving 10,107 patients with intracranial arterial stenosis were selected for the final quantitative analysis. The characteristics of both studies and participants varied substantially. This study reported the pooled incidences of restenosis, TIA, stroke, death, and dissection. Stratified analyses were also conducted according to publication year, country, study design, mean age, proportion of male participants, preprocedural stenosis, lesion location, hypertension, diabetes mellitus, smoking, and study quality. There were no significant differences found between balloon angioplasty and stenting for the risk of restenosis, TIA, stroke, and death, but balloon angioplasty was associated with a higher risk of dissection compared to that seen with stenting. Moreover, subgroup analyses revealed that balloon angioplasty could cause increased risk of TIA and death when the pooled studies were from Eastern countries. Additionally, the risk of death was increased in cases treated with balloon angioplasty in pooled studies of low quality. Further, we noted that balloon angioplasty was associated with an increased risk of dissection when pooled studies had retrospective designs or were of moderate quality.

Several systematic reviews and meta-analyses have already reported patient outcomes following balloon angioplasty or stenting for patients with symptomatic intracranial arterial stenosis. A meta-analysis conducted by Kadooka et al. identified 25 studies and found that balloon angioplasty had similar outcomes to that seen in the stenting arm of the SAMMPRIS study, but balloon angioplasty showed lower rates of late ischemic events and restenosis than stenting did (15). Wang et al. conducted a meta-analysis of 92 studies and found that the risk of short-term and long-term stroke or death was 6.68 and 4.43% after stenting, respectively. They point out that the rate of short-term stroke or death after stenting differ between Western and Eastern countries (16). However, these two studies were based on the outcomes of either balloon angioplasty or stenting, and the results of both procedures were not compared. Therefore, this study was performed to provide an indirect comparison of the treatment effects of balloon angioplasty and stenting for patients with symptomatic intracranial arterial stenosis.

The current study reported comprehensive pooled incidences of restenosis, TIA, stroke, death, and dissection after balloon angioplasty or stenting. While no significant differences were noted between balloon angioplasty and stenting for the risk of restenosis, TIA, stroke, and death, balloon angioplasty was found to be associated with an increased risk of dissection. This could be related to the grade of stenosis, and the use of balloon angioplasty without stenting could induce plaque injury and cause thrombus formation, dissection, or vessel rupture (27, 28). However, there was significant heterogeneity for pooled incidences of restenosis, TIA, stroke, death, and dissection after balloon angioplasty or stenting, and the indirect comparison results could be affected by variant patient characteristics.

Subgroup analyses found several interesting outcomes. We noted that balloon angioplasty was associated with an increased risk of TIA and death when studies were carried out in Eastern countries. These results indicated that stenting was superior to balloon angioplasty in managing symptomatic intracranial arterial stenosis in these countries. These results could be explained by the fact that the prevalence of intracranial arterial stenosis in Eastern countries was higher than those in Western countries. Studies have already illustrated that intracranial arterial stenosis accounts for 10–15% of ischemic strokes in European countries, while causing nearly 54% of ischemic strokes in Asia (29–32). Furthermore, balloon angioplasty was associated with a higher risk of death compared to that seen with stenting when pooled studies were of low quality. This indicates that these results need further verification using high-level evidence. Finally, the risk of dissection was significantly higher after balloon angioplasty than after stenting in studies with retrospective designs or those of moderate quality. These results could be explained by the facts that most studies in this meta-analysis had retrospective designs, that the results from studies of moderate quality were more strongly representative of cohorts, and that the 95%CI of pooled incidence of dissection was narrow.

Several shortcomings of this study should be acknowledged. First, both prospective and retrospective studies were included, and selection, recall, and confounding biases were inevitable. Second, the analysis of this study was based on indirect comparisons, and the characteristics between balloon angioplasty and stenting groups were different. The results need further verification by direct comparison. Moreover, this feature could be explained by the significant heterogeneity across included studies. Third, the studies published between 1995 and 2020, and the develop of device type and medical treatment could affect the prognosis of symptomatic intracranial arterial stenosis. Fourthly, there was significant publication bias owing to the fact that the analysis was based only on published articles. Finally, detailed analyses were restricted because of the use of pooled data.

## Conclusion

This study gives a comprehensive analysis of pooled incidences of restenosis, TIA, stroke, death, and dissection after balloon angioplasty or stenting for patients with symptomatic intracranial arterial stenosis. We noted that balloon angioplasty was associated with a higher risk of dissection than that seen with stenting, whereas no significant differences were detected for risk of restenosis, TIA, stroke, and death. Furthermore, the differences in outcomes after balloon angioplasty and stenting could be affected by country, study design, and study quality. Further direct comparison of balloon angioplasty with stenting for symptomatic intracranial arterial stenosis should be conducted.

## Data Availability Statement

The original contributions presented in the study are included in the article/Supplementary Material, further inquiries can be directed to the corresponding author/s.

## Author Contributions

YS and XL: study design and manuscript drafting, manuscript revising, and final approval. YD: study supervision, data analysis, and result interpretation. BH and JW: literature search and data collection. KM: data curation. YH: software support. All authors have read and confirm that they meet ICMJE criteria for authorship. All contributing authors are aware of and agree to the submission of this manuscript.

## Funding

This study was supported by the Scientific research project of Shanxi Provincial Health and Family Planning Commission (No. 2014015).

## Conflict of Interest

The authors declare that the research was conducted in the absence of any commercial or financial relationships that could be construed as a potential conflict of interest.

## Publisher's Note

All claims expressed in this article are solely those of the authors and do not necessarily represent those of their affiliated organizations, or those of the publisher, the editors and the reviewers. Any product that may be evaluated in this article, or claim that may be made by its manufacturer, is not guaranteed or endorsed by the publisher.
