# Supplementary material for: Balloon Angioplasty vs. Stenting for Symptomatic Intracranial Arterial Stenosis
Source: Front Neurol. 2022 Jun 14;13:878179. doi: 10.3389/fneur.2022.878179 (PMC9237476; doi:10.3389/fneur.2022.878179)
Supplement: Supplementary file 1 [file Data_Sheet_1.docx]

1. ***Table S1. Search strategy in PubMed***

| ***Search step*** | ***Search terms*** |
| --- | --- |
| *#1* | *intracranial atherosclerosis [MeSH] OR intracranial arterial stenosis [All Fields] OR intracranial atherosclerosis [All Fields] OR intracranial stenosis [All Fields] OR intracranial vertebral artery stenosis [All Fields] OR intracranial atherosclerotic stenosis [All Fields] OR intracranial vertebrobasilar artery stenosis [All Fields] OR ischemia cerebrovascular disease caused by artery stenosis [All Fields] OR basilar artery stenosis [All Fields] OR cerebral artery stenosis [All Fields] OR vertebral artery stenosis [All Fields] OR intracranial atherosclerotic diseases [All Fields] OR Atherosclerotic Vertebrobasilar Artery Occlusion [All Fields] OR “intracranial large artery stenoses and occlusions” [All Fields] OR intracranial vertebral artery atherosclerotic stenosis [All Fields] OR vertebral atherosclerotic diseases [All Fields] OR intracranial internal carotid artery stenosis [All Fields] OR atherosclerotic intracranial stenosis[All Fields]* |
| *#2* | *angioplasty [MeSH] OR stenting [All Fields] OR angioplasty [All Fields] OR intracranial stent [All Fields] OR endovascular therapy [All Fields] OR Balloon-Expandable Intracranial Stenting [All Fields] OR “Percutaneous transluminal angioplasty and stenting” [All Fields] OR Endovascular treatment [All Fields] OR balloon-expandable intracranial stent [All Fields] OR percutaneous transluminal angioplasty [All Fields] OR balloon angioplasty [All Fields]* |
| *#3* | *#1 AND #2* |

1. ***Table S2. Baseline characteristics of included studies and involved patients***

| *Study* | *Country* | *Study design* | *Sample size* | *Age (years)* | *Male (%)* | *Preprocedural stenosis, %* | *Lesion location* | *Hypertension (%)* | *DM (%)* | *Smoking (%)* | *Intervention* | *Follow-up* | *Study quality* |
| --- | --- | --- | --- | --- | --- | --- | --- | --- | --- | --- | --- | --- | --- |
| *Touho 1995 [1]* | *Japan* | *Retrospective* | *19* | *65.9* | *57.9* | *83.1* | *ACA* | *NA* | *NA* | *NA* | *BA* | *6.0 months* | *5* |
| *Clark 1995 [2]* | *USA* | *Retrospective* | *17* | *57.0-79.0* | *88.2* | *72.0* | *ICA* | *NA* | *NA* | *NA* | *BA* | *3.0 months* | *5* |
| *Callahan 1997 [3]* | *USA* | *Retrospective* | *15* | *63.1* | *80.0* | *90.1* | *ICA+MCA+VA+BA* | *NA* | *NA* | *NA* | *BA* | *24.0 months* | *5* |
| *Takis 1997 [4]* | *USA* | *Retrospective* | *10* | *64.9* | *50.0* | *89.9* | *ICA+MCA+BA+VA* | *NA* | *NA* | *NA* | *BA* | *12.0 months* | *4* |
| *Mandai 1998 [5]* | *Japan* | *Retrospective* | *11* | *58.5* | *72.7* | *80.0* | *MAC+PCA* | *NA* | *NA* | *NA* | *BA* | *24.0 months* | *4* |
| *Connors 1999 [6]* | *USA* | *Retrospective* | *66* | *NA* | *NA* | *NA* | *ICA+MCA+VA+VBJ+BA+PCA* | *NA* | *NA* | *NA* | *BA* | *12.0 months* | *5* |
| *Eckard 1999 [7]* | *USA* | *Retrospective* | *8* | *49.0-79.0* | *75.0* | *> 80.0* | *ICA* | *NA* | *NA* | *NA* | *BA* | *53.0 months* | *4* |
| *Suh 1999 [8]* | *Korea* | *Retrospective* | *10* | *48.4* | *70.0* | *76.0* | *MCA* | *NA* | *NA* | *NA* | *BA* | *11.0 months* | *5* |
| *Gress 2002 [9]* | *USA* | *Retrospective* | *25* | *50.0-87.0* | *88.0* | *> 70.0* | *VA+VBJ+BA* | *68.0* | *52.0* | *36.0* | *BA* | *12.0 months* | *5* |
| *Terada 2003 [10]* | *Japan* | *Retrospective* | *24* | *66.4/67.1* | *66.7/66.7* | *72.1/75.6* | *ICA* | *NA* | *NA* | *NA* | *BA; stent* | *6.0 months* | *6* |
| *Matsumaru 2004 [11]* | *Japan* | *Retrospective* | *62* | *66.0* | *66.1* | *> 60.0* | *ICA+MCA+VA+BA* | *NA* | *NA* | *NA* | *BA* | *56.0 months* | *5* |
| *Tsuura 2004 [12]* | *Japan* | *Retrospective* | *18* | *62.5* | *77.8* | *80.8* | *VA* | *NA* | *NA* | *NA* | *BA; stent* | *12.0 months* | *5* |
| *Yoon 2005 [13]* | *Korea* | *Retrospective* | *32* | *55.0* | *56.3* | *> 70.0* | *MCA* | *NA* | *NA* | *NA* | *BA* | *20.0 months* | *5* |
| *Henkes 2005 [14]* | *Germany* | *Retrospective* | *15* | *64.0* | *66.7* | *72.0* | *ICA+MCA+VA+BA* | *93.3* | *60.0* | *NA* | *Stent* | *1.0 month* | *4* |
| *Kim 2005 [15]* | *Korea* | *Retrospective* | *17* | *64.0* | *58.8* | *76.1* | *VA+BA* | *100.0* | *41.2* | *41.2* | *Stent* | *21.0 months* | *4* |
| *Lee 2005 [16]* | *Korea* | *Retrospective* | *16* | *59.0* | *68.8* | *67.6* | *MCA* | *NA* | *NA* | *NA* | *Stent* | *10.0 months* | *4* |
| *Lylyk 2005 [17]* | *Argentina* | *Retrospective* | *104* | *67.0* | *NA* | *75.4* | *ICA+MCA+VA+BA+PCA* | *65.3* | *34.6* | *55.7* | *Stent* | *6.0 months* | *5* |
| *Straube 2005 [18]* | *Germany* | *Retrospective* | *11* | *64.0* | *54.5* | *82.0* | *ICA+MCA+VA+BA* | *NA* | *NA* | *NA* | *Stent* | *6.0 months* | *4* |
| *Weber 2005 [19]* | *Germany* | *Retrospective* | *21* | *67.0* | *71.4* | *92.3* | *VA+BA* | *NA* | *NA* | *NA* | *Stent* | *10.0 months* | *5* |
| *Marks 2006 [20]* | *USA* | *Retrospective* | *120* | *62.3* | *70.0* | *82.2* | *ICA+MCA+VA+BA+PCA* | *NA* | *NA* | *NA* | *BA* | *42.3 months* | *6* |
| *Lee 2006 [21]* | *Korea* | *Retrospective* | *10* | *57.0* | *100.0* | *81.0* | *ICA+MCA+BA+VA* | *70.0* | *40.0* | *40.0* | *Stent* | *6.0 months* | *4* |
| *Fiorella 2007 [22]* | *USA* | *Retrospective* | *44* | *64.8* | *79.5* | *82.5* | *VBA* | *NA* | *NA* | *NA* | *Stent* | *43.5 months* | *5* |
| *Freitas 2007 [23]* | *Brazil* | *Retrospective* | *32* | *59.3* | *75.0* | *68.8* | *ICA+MCA+VA+BA* | *NA* | *NA* | *NA* | *Stent* | *10.2 months* | *5* |
| *Jiang 2007 [24]* | *China* | *Prospective* | *79* | *58.5* | *84.8* | *> 50.0* | *VA+BA* | *70.9* | *27.8* | *58.2* | *Stent* | *27.0 months* | *6* |
| *Jiang 2007 [25]* | *China* | *Prospective* | *213* | *52.8* | *82.6* | *> 70.0* | *ICA+MCA+VA+BA* | *63.4* | *20.2* | *65.7* | *Stent* | *26.0 months* | *6* |
| *Steinfort 2007 [26]* | *Australia* | *Retrospective* | *13* | *60.0* | *100.0* | *67.0* | *VA+BA* | *NA* | *NA* | *NA* | *Stent* | *10.9 months* | *4* |
| *Qureshi 2008 [27]* | *USA* | *Retrospective* | *44* | *60.0* | *66.0* | *72.0* | *ICA+MCA+VA+BA+ACA* | *75.0* | *34.5* | *27.5* | *BA; stent* | *12.0 months* | *7* |
| *Siddiq 2008 [28]* | *USA* | *Retrospective* | *193* | *61.9* | *28.5* | *89.6* | *ICA* | *48.7* | *22.3* | *24.4* | *BA; stent* | *60.0 months* | *7* |
| *Suh 2008 [29]* | *Korea* | *Retrospective* | *100* | *61.0* | *78.0* | *69.3* | *ICA+MCA+VA+BA* | *71.0* | *33.0* | *36.0* | *Stent* | *6.0 months* | *5* |
| *Zaidat 2008 [30]* | *USA* | *Prospective* | *129* | *64.2* | *55.0* | *> 70.0* | *ICA+MCA+VA+BA* | *NA* | *NA* | *NA* | *Stent* | *6.0 months* | *6* |
| *Wittkugel 2009 [31]* | *Germany* | *Retrospective* | *42* | *NA* | *NA* | *NA* | *PCA* | *NA* | *NA* | *NA* | *BA* | *26.3 months* | *5* |
| *Seifert 2009 [32]* | *Austria* | *Retrospective* | *17* | *65.9* | *70.6* | *64.3* | *VA+BA* | *NA* | *NA* | *NA* | *Stent* | *24.1 months* | *4* |
| *Wang 2009 [33]* | *China* | *Retrospective* | *16* | *59.2* | *87.5* | *> 70.0* | *BA+VA* | *NA* | *NA* | *NA* | *Stent* | *25.5 months* | *4* |
| *Wolfe 2009 [34]* | *USA* | *Retrospective* | *51* | *63.0* | *59.0* | *> 50.0* | *ICA+MCA+VA+BA+VBJ+PCA* | *NA* | *NA* | *NA* | *Stent* | *14.6 months* | *5* |
| *Zhao 2009 [35]* | *China* | *Retrospective* | *27* | *60.1* | *NA* | *71.8* | *MCA+BA+VA* | *NA* | *NA* | *NA* | *Stent* | *1.0 month* | *5* |
| *Costalat 2010 [36]* | *France* | *Retrospective* | *42* | *62.9* | *88.1* | *73.9* | *VA+BA+ICA+MCA* | *NA* | *NA* | *NA* | *BA; stent* | *6.0 months* | *6* |
| *Blasel 2010 [37]* | *Germany* | *Retrospective* | *50* | *62.6* | *85.0* | *76.2* | *ICA* | *NA* | *NA* | *NA* | *Stent* | *24.0 months* | *5* |
| *Chamczuk 2010 [38]* | *USA* | *Retrospective* | *66* | *62.7* | *63.6* | *75.5* | *ICA+MCA+VA+BA* | *NA* | *NA* | *NA* | *Stent* | *12.0 months* | *5* |
| *Jiang 2010 [39]* | *China* | *Prospective* | *139* | *58.5* | *92.1* | *> 50.0* | *VA+BA* | *78.4* | *28.8* | *66.2* | *Stent* | *1.0 month* | *6* |
| *Kurre 2010 [40]* | *Germany* | *Prospective* | *372* | *NA* | *75.8* | *> 50.0* | *ICA+MCA+VA+BA* | *84.4* | *39.2* | *22.8* | *Stent* | *12.0 months* | *6* |
| *Lanfranconi 2010 [41]* | *Italy* | *Retrospective* | *34* | *62.3* | *91.2* | *> 70.0* | *ICA+MCA+VA+BA* | *76.5* | *35.3* | *14.7* | *Stent* | *1.0 month* | *5* |
| *Povedano 2010 [42]* | *Argentina* | *Retrospective* | *25* | *63.0* | *84.0* | *> 50.0* | *VA+BA+PCA* | *84.0* | *24.0* | *40.0* | *Stent* | *12.0 months* | *6* |
| *Vajda 2010 [43]* | *Germany* | *Retrospective* | *25* | *67.1* | *80.0* | *61.0* | *ICA+MCA+VA+BA* | *84.0* | *52.0* | *68.0* | *Stent* | *12.0 months* | *5* |
| *Nguyen 2011 [44]* | *USA* | *Retrospective* | *74* | *62.0* | *55.0* | *78.7* | *ICA+BA+VA+ICA+PCA* | *82.0* | *42.0* | *28.0* | *BA* | *3.0 months* | *6* |
| *Costalat 2011 [45]* | *France* | *Retrospective* | *60* | *65.3* | *66.7* | *80.2* | *ICA+MCA+VA+BA* | *NA* | *NA* | *NA* | *Stent* | *12.0 months* | *5* |
| *Fields 2011 [46]* | *USA* | *Retrospective* | *26* | *66.0* | *69.2* | *80.0* | *ICA+MCA+VA+BA* | *96.2* | *26.9* | *26.9* | *Stent* | *14.0 months* | *5* |
| *Fiorella 2011 [47]* | *USA* | *Prospective* | *158* | *62.7* | *60.1* | *> 50.0* | *ICA* | *NA* | *NA* | *NA* | *Stent* | *21.0 months* | *6* |
| *Guo 2011 [48]* | *China* | *Retrospective* | *53* | *58.0* | *64.1* | *76.5* | *MCA* | *85.3* | *NA* | *39.6* | *Stent* | *6.0 months* | *5* |
| *Jiang 2011 [49]* | *China* | *Prospective* | *100* | *53.2* | *87.0* | *> 70.0* | *ICA+MCA+VA+BA* | *72.0* | *32.0* | *68.0* | *Stent* | *21.6 months* | *6* |
| *Li 2011 [50]* | *China* | *Retrospective* | *47* | *51.7* | *72.3* | *> 70.0* | *MCA* | *38.0* | *40.0* | *40.0* | *Stent* | *12.9 months* | *5* |
| *Liu 2011 [51]* | *China* | *Retrospective* | *111* | *60.0* | *57.7* | *> 70.0* | *ICA+MCA+VA+BA* | *NA* | *NA* | *NA* | *Stent* | *12.0 months* | *6* |
| *Lu 2011 [52]* | *Korea* | *Retrospective* | *130* | *NA* | *71.0* | *> 70.0* | *ICA+MCA+VA+BA* | *75.0* | *44.0* | *35.0* | *Stent* | *6.0 months* | *6* |
| *Park 2011 [53]* | *Korea* | *Retrospective* | *12* | *65.9* | *58.3* | *80.4* | *ICA+MCA+VA* | *NA* | *NA* | *NA* | *Stent* | *12.5 months* | *4* |
| *Tang 2011 [54]* | *China* | *Retrospective* | *53* | *66.1* | *83.0* | *> 70.0* | *ICA+MCA+VA+BA+ACA* | *79.2* | *47.2* | *32.1* | *Stent* | *17.3 months* | *7* |
| *Yu 2011 [55]* | *China* | *Prospective* | *60* | *64.3* | *78.3* | *78.4* | *ICA+MCA+VA+BA* | *NA* | *NA* | *NA* | *Stent* | *12.0 months* | *5* |
| *Yue 2011 [56]* | *China* | *Retrospective* | *67* | *64.3* | *78.3* | *76.0* | *MCA* | *82.1* | *41.8* | *17.9* | *Stent* | *36.0 months* | *7* |
| *Chavent 2012 [57]* | *France* | *Retrospective* | *21* | *65.0* | *90.5* | *69.0* | *ICA+MCA+VA+BA* | *NA* | *NA* | *NA* | *Stent* | *16.8 months* | *4* |
| *Dorn 2012 [58]* | *Germany* | *Retrospective* | *49* | *68.6* | *71.4* | *> 70.0* | *ICA+MCA+VA+BA* | *NA* | *NA* | *NA* | *Stent* | *13.0 months* | *5* |
| *Kim 2012 [59]* | *Korea* | *Retrospective* | *77* | *60.8* | *50.6* | *72.3* | *ICA+MCA+VA+BA* | *NA* | *NA* | *NA* | *Stent* | *29.4 months* | *5* |
| *Lee 2012 [60]* | *Korea* | *Retrospective* | *28* | *NA* | *NA* | *83.3* | *ICA* | *NA* | *NA* | *NA* | *Stent* | *14.4 months* | *4* |
| *Li 2012 [61]* | *China* | *Retrospective* | *30* | *59.0* | *83.3* | *82.3* | *VA+BA* | *86.7* | *36.7* | *46.7* | *Stent* | *17.8 months* | *4* |
| *Lee 2012 [62]* | *Korea* | *Retrospective* | *19* | *65.0* | *63.2* | *> 50.0* | *ICA+MCA+VA+BA* | *NA* | *NA* | *NA* | *Stent* | *13.2 months* | *4* |
| *Miao 2012 [63]* | *China* | *Prospective* | *36* | *53.4* | *66.7* | *83.9* | *MCA* | *63.9* | *22.2* | *58.3* | *Stent* | *9.8 months* | *7* |
| *Mohammadian 2012 [64]* | *Iran* | *Prospective* | *34* | *67.9* | *58.8* | *> 70.0* | *ICA+MCA* | *70.6* | *70.6* | *35.2* | *Stent* | *15.2 months* | *7* |
| *Tarlov 2012 [65]* | *USA* | *Retrospective* | *41* | *65.0* | *73.0* | *> 50.0* | *ICA+MCA+VA+BA* | *90.0* | *53.0* | *NA* | *Stent* | *24.0 months* | *5* |
| *Vajda 2012 [66]* | *Germany* | *Retrospective* | *95* | *68.0* | *83.2* | *> 50.0* | *ICA+VA+BA* | *NA* | *NA* | *NA* | *Stent* | *12.0 months* | *5* |
| *Vajda 2012 [67]* | *Germany* | *Retrospective* | *189* | *68.0* | *69.8* | *65.4* | *ICA+MCA+VA+BA+PCA* | *NA* | *NA* | *NA* | *Stent* | *10.2 months* | *6* |
| *Yu 2012 [68]* | *China* | *Prospective* | *57* | *64.2* | *78.9* | *72.8* | *ICA+MCA+VA+BA* | *NA* | *NA* | *NA* | *Stent* | *1.0 month* | *6* |
| *Zhang 2012 [69]* | *China* | *Retrospective* | *50* | *56.7* | *75.5* | *73.9* | *ICA+MCA+VA+BA* | *NA* | *NA* | *NA* | *Stent* | *12.0 months* | *5* |
| *Tomycz 2013 [70]* | *USA* | *Retrospective* | *26* | *63.0* | *69.2* | *71.2* | *BA+ICA+MCA+VA+VBJ* | *84.6* | *34.6* | *NA* | *BA* | *12.0 months* | *5* |
| *Alurkar 2013 [71]* | *India* | *Retrospective* | *182* | *> 18.0* | *61.0* | *> 70.0* | *ICA+MCA+BA+VA* | *NA* | *NA* | *NA* | *Stent* | *12.0 months* | *5* |
| *Aparici Robles 2013 [72]* | *Spain* | *Retrospective* | *26* | *63.0* | *80.8* | *> 50.0* | *ICA+MCA+BA+VA* | *NA* | *NA* | *NA* | *Stent* | *46.0 months* | *4* |
| *Gandini 2013 [73]* | *Italy* | *Prospective* | *21* | *70.5* | *57.1* | *84.0* | *ICA+MCA+BA+VA* | *NA* | *NA* | *NA* | *Stent* | *19.5 months* | *5* |
| *Lee 2013 [74]* | *Korea* | *Retrospective* | *31* | *62.9* | *67.6* | *79.0* | *MCA* | *NA* | *NA* | *NA* | *Stent* | *7.0 months* | *5* |
| *Lu 2013 [75]* | *China* | *Retrospective* | *24* | *65.0* | *58.3* | *77.1* | *VA+BA* | *83.0* | *50.0* | *NA* | *Stent* | *35.0 months* | *5* |
| *Park 2013 [76]* | *Korea* | *Retrospective* | *115* | *59.8* | *72.2* | *> 70.0* | *ICA+MCA+VA+BA* | *73.9* | *38.3* | *34.8* | *Stent* | *6.0 months* | *7* |
| *Park 2013 [77]* | *Korea* | *Retrospective* | *11* | *59.0* | *100.0* | *67.0* | *ICA+MCA+VA+BA* | *NA* | *NA* | *NA* | *Stent* | *55.0 months* | *4* |
| *Rohde 2013 [78]* | *Germany* | *Prospective* | *100* | *64.0* | *74.0* | *83.0* | *ICA+MCA+VA+BA* | *NA* | *NA* | *NA* | *Stent* | *1.0 month* | *5* |
| *Zhang 2013 [79]* | *China* | *Retrospective* | *61* | *57.8* | *78.7* | *76.8* | *MCA* | *59.0* | *21.3* | *42.6* | *Stent* | *12.0 months* | *5* |
| *Chang 2014 [80]* | *Korea* | *Retrospective* | *11* | *53.0* | *72.7* | *83.6* | *MCA* | *NA* | *NA* | *NA* | *BA* | *12.0 months* | *4* |
| *Derdeyn 2014 [81]* | *USA* | *Prospective* | *224* | *61.0* | *57.0* | *> 70.0* | *ICA+MCA+BA+VA* | *89.0* | *47.0* | *24.0* | *Stent* | *32.4 months* | *7* |
| *Silber 2014 [82]* | *Germany* | *Retrospective* | *80* | *69.5* | *72.5* | *> 70.0* | *ICA+MCA+BA+VA* | *92.5* | *41.3* | *25.0* | *Stent* | *1.0 month* | *6* |
| *Yu 2014 [83]* | *China* | *Prospective* | *65* | *62.9* | *74.6* | *71.8* | *MCA+BA+VA* | *NA* | *NA* | *NA* | *Stent* | *12.0 months* | *5* |
| *Miao 2015 [84]* | *China* | *Prospective* | *120* | *59.3* | *77.5* | *82.0* | *ICA+MCA+VA+BA* | *69.0* | *33.5* | *59.5* | *BA; stent* | *1.0 month* | *7* |
| *Okada 2015 [85]* | *Japan* | *Retrospective* | *47* | *65.7* | *70.2* | *> 70.0* | *MCA* | *NA* | *NA* | *NA* | *BA* | *51.5 months* | *5* |
| *Feng 2015 [86]* | *China* | *Retrospective* | *44* | *60.5* | *72.7* | *79.3* | *MCA+ACA+PCA+BA+VA* | *68.2* | *29.6* | *25.0* | *Stent* | *1.0 month* | *5* |
| *Li 2015 [87]* | *China* | *Prospective* | *433* | *57.3* | *69.1* | *82.3* | *ICA+MCA+VA+BA* | *68.1* | *28.9* | *33.9* | *Stent* | *69.0 months* | *6* |
| *Miao 2015 [88]* | *China* | *Prospective* | *300* | *58.3* | *76.0* | *> 70.0* | *ICA+MCA+VA+BA* | *71.7* | *30.0* | *26.0* | *Stent* | *1.0 month* | *7* |
| *von Schoenfeldt 2015 [89]* | *Germany* | *Retrospective* | *92* | *67.0* | *79.0* | *> 70.0* | *ICA+MCA+VA+BA* | *NA* | *NA* | *NA* | *Stent* | *1.0 month* | *5* |
| *Wang 2015 [90]* | *China* | *Prospective* | *88* | *62.6* | *87.5* | *84.9* | *VA* | *77.3* | *52.3* | *34.1* | *Stent* | *29.3 months* | *6* |
| *Xiong 2015 [91]* | *China* | *Prospective* | *376* | *59.1* | *74.2* | *> 70.0* | *ICA+MCA+VA+BA* | *NA* | *NA* | *NA* | *Stent* | *1.0 month* | *6* |
| *Yin 2015 [92]* | *China* | *Retrospective* | *48* | *59.2* | *75.0* | *> 70.0* | *ICA+MCA+ACA+PCA+BA+VA* | *72.9* | *37.5* | *45.8* | *Stent* | *12.0 months* | *5* |
| *Zaidat 2015 [93]* | *USA* | *Prospective* | *58* | *61.8* | *70.7* | *> 70.0* | *ICA* | *84.5* | *43.1* | *19.0* | *Stent* | *12.0 months* | *7* |
| *Al Said 2016 [94]* | *Kingdom of Saudi Arabia* | *Retrospective* | *19* | *58.3* | *73.4* | *> 50.0* | *ICA+MCA+BA+VA* | *NA* | *NA* | *NA* | *Stent* | *6.0 months* | *4* |
| *Bai 2016 [95]* | *China* | *Prospective* | *91* | *61.3* | *72.5* | *82.2* | *BA* | *86.8* | *39.6* | *37.4* | *Stent* | *31.3 months* | *6* |
| *Cheng 2016 [96]* | *China* | *Retrospective* | *583* | *58.1* | *85.6* | *> 70.0* | *ICA+MCA+BA+VA* | *72.2* | *37.2* | *43.6* | *Stent* | *3.0 months* | *6* |
| *Duan 2016 [97]* | *China* | *Retrospective* | *44* | *62.5* | *79.5* | *> 70.0* | *ICA+MCA+ACA+PCA+BA+VA* | *79.6* | *31.8* | *25.0* | *Stent* | *25.5 months* | *5* |
| *Gao 2016 [98]* | *China* | *Prospective* | *100* | *56.0* | *73.0* | *82.7* | *ICA+MCA+BA+VA* | *61.0* | *25.0* | *44.0* | *Stent* | *1.0 month* | *6* |
| *Liu 2016 [99]* | *China* | *Prospective* | *97* | *58.4* | *83.5* | *> 70.0* | *BA+VA* | *82.5* | *28.9* | *61.9* | *Stent* | *1.0 month* | *6* |
| *Wang 2016 [100]* | *China* | *Retrospective* | *60* | *56.8* | *70.0* | *76.3* | *ICA+MCA+BA+VA* | *NA* | *NA* | *NA* | *Stent* | *6.2 months* | *5* |
| *Wang 2016 [101]* | *China* | *Prospective* | *58* | *55.1* | *48.3* | *84.3* | *ICA+MCA+BA+VA* | *72.4* | *27.6* | *17.2* | *Stent* | *12.0 months* | *5* |
| *Wang 2016 [102]* | *China* | *Retrospective* | *196* | *53.0* | *70.9* | *80.6* | *MCA* | *53.6* | *13.3* | *37.2* | *Stent* | *30.0 months* | *6* |
| *Zhao 2016 [103]* | *China* | *Prospective* | *278* | *60.1* | *82.0* | *82.5* | *MCA* | *62.6* | *21.2* | *51.4* | *Stent* | *24.0 months* | *6* |
| *Zhang 2016 [104]* | *China* | *Retrospective* | *102* | *64.0* | *86.3* | *> 70.0* | *ICA* | *68.6* | *56.9* | *25.5* | *Stent* | *74.0 months* | *5* |
| *Markus 2017 [105]* | *UK* | *Prospective* | *91* | *68.3* | *80.0* | *> 50.0* | *VA* | *73.0* | *22.0* | *20.0* | *Stent* | *12.0 months* | *5* |
| *Yeo 2017 [106]* | *China* | *Retrospective* | *50* | *64.8* | *22.0* | *76.5* | *ICA+MCA+BA+VA* | *78.0* | *30.0* | *NA* | *Stent* | *12.0 months* | *5* |
| *Ueda 2018 [107]* | *Japan* | *Retrospective* | *72* | *58.9* | *71.0* | *81.9* | *MCA* | *76.0* | *21.0* | *NA* | *BA* | *63.0 months* | *5* |
| *Gruber 2018 [108]* | *Switzerland* | *Retrospective* | *11* | *67.0* | *45.0* | *80.0* | *ICA+MCA+BA+VA* | *73.0* | *36.0* | *18.0* | *Stent* | *10.0 months* | *6* |
| *Maier 2018 [109]* | *Germany* | *Retrospective* | *79* | *70.0* | *76.0* | *> 50.0* | *BA* | *93.7* | *34.2* | *25.3* | *Stent* | *6.0 months* | *7* |
| *Yu 2018 [110]* | *China* | *Retrospective* | *36* | *60.0* | *72.2* | *71.4* | *ICA+MCA+BA+VA* | *75.0* | *25.0* | *NA* | *Stent* | *84.4 months* | *5* |
| *Zhao 2018 [111]* | *China* | *Retrospective* | *576* | *55.0* | *62.2* | *> 70.0* | *ICA+MCA+BA+VA* | *67.7* | *42.7* | *42.7* | *Stent* | *1.0 month* | *6* |
| *Wang 2018 [112]* | *China* | *Retrospective* | *35* | *64.3* | *74.3* | *88.4* | *ICA+MCA+BA+VA* | *62.8* | *25.7* | *NA* | *BA* | *9.7 months* | *5* |
| *Baik 2018 [113]* | *Korea* | *Retrospective* | *34* | *68.0* | *44.1* | *77.0* | *MCA* | *52.9* | *26.5* | *38.2* | *Stent* | *67.5 months* | *5* |
| *Peng 2019 [114]* | *China* | *Retrospective* | *133* | *59.1* | *78.2* | *81.9* | *ICA+MCA+VA+BA* | *82.0* | *42.2* | *26.3* | *BA* | *12.0 months* | *6* |
| *Zhou 2019 [115]* | *China* | *Retrospective* | *213* | *57.4* | *68.5* | *86.3* | *VA* | *74.2* | *38.0* | *25.8* | *Stent* | *3.0 months* | *6* |
| *Salik 2019 [116]* | *Turkey* | *Retrospective* | *68* | *62.0* | *82.0* | *> 70.0* | *ICA+MCA+VA+BA* | *NA* | *NA* | *NA* | *Stent* | *22.0 months* | *5* |
| *Zhang 2019 [117]* | *China* | *Retrospective* | *167* | *59.7* | *78.4* | *82.8* | *VA+BA* | *80.2* | *34.7* | *22.2* | *Stent* | *3.0 months* | *7* |
| *Gross 2019 [118]* | *USA* | *Prospective* | *60* | *66.0* | *55.0* | *> 70.0* | *ICA+MCA+VA+BA* | *77.0* | *22.0* | *33.0* | *Stent* | *3.0 months* | *5* |
| *Park 2020 [119]* | *Korea* | *Retrospective* | *95* | *66.5* | *67.4* | *76.8* | *ICA+MCA+VA* | *NA* | *NA* | *NA* | *Stent* | *34.9 months* | *5* |
| *Zhang 2020 [120]* | *China* | *Prospective* | *115* | *59.2* | *76.5* | *84.2* | *ICA+MCA+VA+BA* | *46.1* | *13.9* | *28.7* | *Stent* | *24.0 months* | *7* |

1. ***Reference lists***
2. *Touho H. Percutaneous transluminal angioplasty in the treatment of atherosclerotic disease of the anterior cerebral circulation and hemodynamic evaluation. J Neurosurg. 1995;82(6):953-60.*
3. *Clark WM, Barnwell SL, Nesbit G, et al. Safety and efficacy of percutaneous transluminal angioplasty for intracranial atherosclerotic stenosis. Stroke. 1995;26(7): 1200-4.*
4. *Callahan AS 3rd, Berger BL. Balloon angioplasty of intracranial arteries for stroke prevention. J Neuroimaging. 1997;7(4):232-5.*
5. *Takis C, Kwan ES, Pessin MS, et al. Intracranial angioplasty: experience and complications. AJNR Am J Neuroradiol. 1997;18(9):1661-8.*
6. *Mandai S, Matsushita H, Akamatsu S, et al. Percutaneous transluminal angioplasty for intracranial atherosclerotic stenosis. Interv Neuroradiol. 1998;4 Suppl 1:53-6.*
7. *Connors JJ 3rd, Wojak JC. Percutaneous transluminal angioplasty for intracranial atherosclerotic lesions: evolution of technique and short-term results. J Neurosurg. 1999;91(3):415-23.*
8. *Eckard DA, Zarnow DM, McPherson CM, et al. Intracranial internal carotid artery angioplasty: technique with clinical and radiographic results and follow-up. AJR Am J Roentgenol. 1999;172(3):703-7.*
9. *Suh DC, Sung KB, Cho YS, et al. Transluminal angioplasty for middle cerebral artery stenosis in patients with acute ischemic stroke. AJNR Am J Neuroradiol. 1999; 20(4):553-8.*
10. *Gress DR, Smith WS, Dowd CF,* *et al. Angioplasty for intracranial symptomatic vertebrobasilar ischemia. Neurosurgery. 2002;51(1):23-7; discussion 27-9.*
11. *Terada T, Tsuura M, Matsumoto H, et al. Endovascular therapy for stenosis of the petrous or cavernous portion of the internal carotid artery: percutaneous transluminal angioplasty compared with stent placement. J Neurosurg. 2003; 98(3):491-7.*
12. *Matsumaru Y, Tsuruta W, Takigawa T, et al. Percutaneous transluminal angioplasty for atherosclerotic stenoses of intracranial vessels. Interv Neuroradiol. 2004;10 Suppl 2(Suppl 2):17-20.*
13. *Tsuura M, Terada T, Masuo O, et al. Clinical results of percutaneous transluminal angioplasty and stenting for intracranial vertebrobasilar atherosclerotic stenoses and occlusions. Interv Neuroradiol. 2004;10 Suppl 2(Suppl 2):21-5.*
14. *Yoon W, Seo JJ, Cho KH, et al. Symptomatic middle cerebral artery stenosis treated with intracranial angioplasty: experience in 32 patients. Radiology. 2005; 237(2):620-6.*
15. *Henkes H, Miloslavski E, Lowens S, et al. Treatment of intracranial atherosclerotic stenoses with balloon dilatation and self-expanding stent deployment (WingSpan). Neuroradiology. 2005;47(3):222-8.*
16. *Kim DJ, Lee BH, Kim DI, et al. Stent-assisted angioplasty of symptomatic intracranial vertebrobasilar artery stenosis: feasibility and follow-up results. AJNR Am J Neuroradiol. 2005;26(6):1381-8.*
17. *Lee TH, Kim DH, Lee BH, et al. Preliminary results of endovascular stent-assisted angioplasty for symptomatic middle cerebral artery stenosis. AJNR Am J Neuroradiol. 2005;26(1):166-74.*
18. *Lylyk P, Vila JF, Miranda C, et al. Endovascular reconstruction by means of stent placement in symptomatic intracranial atherosclerotic stenosis. Neurol Res. 2005;27 Suppl 1:S84-8.*
19. *Straube T, Stingele R, Jansen O. Primary stenting of intracranial atherosclerotic stenoses. Cardiovasc Intervent Radiol. 2005 May-Jun;28(3):289-95.*
20. *Weber W, Mayer TE, Henkes H, et al. Stent-angioplasty of intracranial vertebral and basilar artery stenoses in symptomatic patients. Eur J Radiol. 2005;55(2):231-6.*
21. *Marks MP, Wojak JC, Al-Ali F, et al. Angioplasty for symptomatic intracranial stenosis: clinical outcome. Stroke. 2006;37(4):1016-20.*
22. *Lee CY, Yim MB. Primary stent therapy for symptomatic intracranial atherosclerotic stenosis: 1-year follow-up angiographic and midterm clinical outcomes. J Neurosurg. 2006;105(2):235-41.*
23. *Fiorella D, Chow MM, Anderson M, et al. A 7-year experience with balloon-mounted coronary stents for the treatment of symptomatic vertebrobasilar intracranial atheromatous disease. Neurosurgery. 2007;61(2):236-42; discussion 242-3.*
24. *Freitas JM, Zenteno M, Aburto-Murrieta Y, et al. Intracranial arterial stenting for symptomatic stenoses: a Latin American experience. Surg Neurol. 2007; 68(4):378-86.*
25. *Jiang WJ, Xu XT, Du B, et al. Long-term outcome of elective stenting for symptomatic intracranial vertebrobasilar stenosis. Neurology. 2007;68(11):856-8.*
26. *Jiang WJ, Xu XT, Du B, et al. Comparison of elective stenting of severe vs moderate intracranial atherosclerotic stenosis. Neurology. 2007;68(6):420-6.*
27. *Steinfort B, Ng PP, Faulder K, et al. Midterm outcomes of paclitaxel-eluting stents for the treatment of intracranial posterior circulation stenoses. J Neurosurg. 2007;106(2):222-5.*
28. *Qureshi AI, Hussein HM, El-Gengaihy A, et al. Concurrent comparison of outcomes of primary angioplasty and of stent placement in high-risk patients with symptomatic intracranial stenosis. Neurosurgery. 2008;62(5):1053-60; discussion 1060-2.*
29. *Siddiq F, Vazquez G, Memon MZ, et al. Comparison of primary angioplasty with stent placement for treating symptomatic intracranial atherosclerotic diseases: a multicenter study. Stroke. 2008;39(9):2505-10.*
30. *Suh DC, Kim JK, Choi JW, et al. Intracranial stenting of severe symptomatic intracranial stenosis: results of 100 consecutive patients. AJNR Am J Neuroradiol. 2008;29(4):781-5.*
31. *Zaidat OO, Klucznik R, Alexander MJ, et al. The NIH registry on use of the Wingspan stent for symptomatic 70-99% intracranial arterial stenosis. Neurology. 2008;70(17):1518-24.*
32. *Wittkugel O, Rosenkranz M, Burckhardt D, et al. Langzeitergebnisse nach endovaskulärer Behandlung von Hochrisiko-Patienten mit mehrfach symptomatischen intrakraniellen Stenosen der vertebrobasilären Strombahn [Long-term outcome after endovascular treatment of high-risk patients with recurrently symptomatic intracranial stenoses of the posterior circulation]. Rofo. 2009;181(8):782-91.*
33. *Seifert T, Augustin M, Klein GE, et al. Symptomatic stenosis of the vertebrobasilar arteries: results of extra- and intracranial stent-PTA. Eur J Neurol. 2009;16(1):31-6.*
34. *Wang B, Miao ZR, Li GL, et al. Treatment of symptomatic complex posterior circulation cerebral artery stenosis with balloon-mounted stents: technique feasibility and outcome. Neuroradiology. 2009;51(5):319-26.*
35. *Wolfe TJ, Fitzsimmons BF, Hussain SI, et al. Long term clinical and angiographic outcomes with the Wingspan stent for treatment of symptomatic 50-99% intracranial atherosclerosis: single center experience in 51 cases. J Neurointerv Surg. 2009; 1(1):40-3.*
36. *Zhao ZW, Deng JP, He SM, et al. Intracranial angioplasty with Gateway-Wingspan system for symptomatic atherosclerotic stenosis: preliminary results of 27 Chinese patients. Surg Neurol. 2009;72(6):607-11; discussion 611.*
37. *Costalat V, Maldonado IL, Zerlauth JB, et al. Endovascular treatment of symptomatic intracranial arterial stenosis: six-year experience in a single-center series of 42 consecutive patients with acute and mid-term results. Neurosurgery. 2010; 67(6):1505-13; discussion 1513-4.*
38. *Blasel S, Yükzek Z, Kurre W, et al. Recanalization results after intracranial stenting of atherosclerotic stenoses. Cardiovasc Intervent Radiol. 2010;33(5):914-20.*
39. *Chamczuk AJ, Ogilvy CS, Snyder KV, et al. Elective stenting for intracranial stenosis under conscious sedation. Neurosurgery. 2010;67(5):1189-93; discussion 1194.*
40. *Jiang WJ, Du B, Hon SF, et al. Do patients with basilar or vertebral artery stenosis have a higher stroke incidence poststenting? J Neurointerv Surg. 2010;2(1): 50-4.*
41. *Kurre W, Berkefeld J, Brassel F, et al. In-hospital complication rates after stent treatment of 388 symptomatic intracranial stenoses: results from the INTRASTENT multicentric registry. Stroke. 2010;41(3):494-8.*
42. *Lanfranconi S, Bersano A, Branca V, et al. Stenting for the treatment of high-grade intracranial stenoses. J Neurol. 2010;257(11):1899-908.*
43. *Povedano G, Zuberbuhler P, Lylyk P, et al. Management strategies in posterior circulation intracranial atherosclerotic disease. J Endovasc Ther. 2010;17(3):308-13.*
44. *Vajda Z, Miloslavski E, Güthe T, et al. Treatment of intracranial atherosclerotic arterial stenoses with a balloon-expandable cobalt chromium stent (Coroflex Blue): procedural safety, efficacy, and midterm patency. Neuroradiology. 2010;52(7): 645-51.*
45. *Nguyen TN, Zaidat OO, Gupta R, et al. Balloon angioplasty for intracranial atherosclerotic disease: periprocedural risks and short-term outcomes in a multicenter study. Stroke. 2011;42(1):107-11.*
46. *Costalat V, Maldonado IL, Vendrell JF, et al. Endovascular treatment of symptomatic intracranial stenosis with the Wingspan stent system and Gateway PTA balloon: a multicenter series of 60 patients with acute and midterm results. J Neurosurg. 2011;115(4):686-93.*
47. *Fields JD, Petersen BD, Lutsep HL, et al. Drug eluting stents for symptomatic intracranial and vertebral artery stenosis. Interv Neuroradiol. 2011;17(2):241-7.*
48. *Fiorella DJ, Turk AS, Levy EI, et al. U.S. Wingspan Registry: 12-month follow-up results. Stroke. 2011;42(7):1976-81.*
49. *Guo XB, Ma N, Hu XB, et al. Wingspan stent for symptomatic M1 stenosis of middle cerebral artery. Eur J Radiol. 2011;80(3):e356-60.*
50. *Jiang WJ, Yu W, Du B, et al. Outcome of patients with ≥70% symptomatic intracranial stenosis after Wingspan stenting. Stroke. 2011;42(7):1971-5.*
51. *Li J, Zhao ZW, Gao GD, et al. Wingspan stenting with modified predilation for symptomatic middle cerebral artery stenosis. Catheter Cardiovasc Interv. 2011;78(2): 286-93.*
52. *Liu X, Wang W, Tang Z, et al. Clinical study of PTAS therapy for patients with ischemia cerebrovascular disease caused by artery stenosis. J Huazhong Univ Sci Technolog Med Sci. 2011;31(1):67-72.*
53. *Lü PH, Park JW, Park S, et al. Intracranial stenting of subacute symptomatic atherosclerotic occlusion versus stenosis. Stroke. 2011;42(12):3470-6.*
54. *Park TS, Choi BJ, Lee TH, et al. Urgent recanalization with stenting for severe intracranial atherosclerosis after transient ischemic attack or minor stroke. J Korean Neurosurg Soc. 2011;50(4):322-6.*
55. *Tang CW, Chang FC, Chern CM, et al. Stenting versus medical treatment for severe symptomatic intracranial stenosis. AJNR Am J Neuroradiol. 2011;32(5):911-6.*
56. *Yu SC, Leung TW, Lee KT, et al. Angioplasty and stenting of atherosclerotic middle cerebral arteries with Wingspan: evaluation of clinical outcome, restenosis, and procedure outcome. AJNR Am J Neuroradiol. 2011;32(4):753-8.*
57. *Yue X, Yin Q, Xi G, et al. Comparison of BMSs with SES for symptomatic intracranial disease of the middle cerebral artery stenosis. Cardiovasc Intervent Radiol. 2011;34(1):54-60.*
58. *Chavent A, Kazemi A, Voguet C, et al. Endovascular treatment of symptomatic intracranial atheromatous stenosis: a single center study of 21 consecutive cases. J Neuroradiol. 2012;39(5):332-41.*
59. *Dorn F, Prothmann S, Wunderlich S, et al. Stent angioplasty of intracranial stenosis: single center experience of 54 cases. Clin Neuroradiol. 2012;22(2):149-56.*
60. *Kim KS, Hwang DH, Ko YH, et al. Usefulness of stent implantation for treatment of intracranial atherosclerotic stenoses. Neurointervention. 2012;7(1):27-33.*
61. *Lee T, Song S, Lee S. Urgent recanalization with stent for severe intracranial stenosis in patients with transient ischemic attack or minor stroke. J Neurointervent Surg 2012; 4(1): E-007.*
62. *Li J, Zhao ZW, Gao GD, et al. Wingspan stent for high-grade symptomatic vertebrobasilar artery atherosclerotic stenosis. Cardiovasc Intervent Radiol. 2012; 35(2):268-78.*
63. *Lee JH, Yun JK, Kim DW, et al. Clinical and angiographic outcomes of wingspan stent placement for treatment of symptomatic intracranial stenosis: single center experience with 19 cases. J Cerebrovasc Endovasc Neurosurg. 2012;14(3):157-63.*
64. *Miao Z, Jiang L, Wu H, et al. Randomized controlled trial of symptomatic middle cerebral artery stenosis: endovascular versus medical therapy in a Chinese population. Stroke. 2012;43(12):3284-90.*
65. *Mohammadian R, Pashapour A, Sharifipour E, et al. A Comparison of Stent Implant versus Medical Treatment for Severe Symptomatic Intracranial Stenosis: A Controlled Clinical Trial. Cerebrovasc Dis Extra. 2012;2(1):108-20.*
66. *Tarlov N, Jahan R, Saver JL, et al. Treatment of high risk symptomatic intracranial atherosclerosis with balloon mounted coronary stents and Wingspan stents: single center experience over a 10 year period. J Neurointerv Surg. 2012;4(1): 34-9.*
67. *Vajda Z, Aguilar M, Göhringer T, et al. Treatment of intracranial atherosclerotic disease with a balloon-expandable paclitaxel eluting stent: procedural safety, efficacy and mid-term patency. Clin Neuroradiol. 2012;22(3):227-33.*
68. *Vajda Z, Schmid E, Güthe T, et al. The modified Bose method for the endovascular treatment of intracranial atherosclerotic arterial stenoses using the Enterprise stent. Neurosurgery. 2012;70(1):91-101; discussion 101.*
69. *Yu SC, Leung TW, Hung EH, et al. Angioplasty and stenting for intracranial atherosclerotic stenosis with nitinol stent: factors affecting technical success and patient safety. Neurosurgery. 2012;70(1 Suppl Operative):104-13.*
70. *Zhang L, Huang Q, Zhang Y, et al. Wingspan stents for the treatment of symptomatic atherosclerotic stenosis in small intracranial vessels: safety and efficacy evaluation. AJNR Am J Neuroradiol. 2012;33(2):343-7.*
71. *Tomycz L, Bansal NK, Lockney T, et al. Primary balloon angioplasty for symptomatic, high-grade intracranial stenosis. Surg Neurol Int. 2013;4:18.*
72. *Alurkar A, Karanam LS, Oak S, et al. Role of balloon-expandable stents in intracranial atherosclerotic disease in a series of 182 patients. Stroke. 2013;44(7): 2000-3.*
73. *Aparici Robles F, Mainar Tello E, Vázquez-Añón V, et al. Tratamiento endovascular de estenosis intracraneales sintomáticas: resultados a corto y largo plazo de un único centro [Endovascular treatment of symptomatic intracranial stenoses: short- and long-term results in a single center]. Radiologia. 2013;55(5): 416-21.*
74. *Gandini R, Chiaravalloti A, Pampana E, et al. Intracranial atheromatous disease treatment with the Wingspan stent system: evaluation of clinical, procedural outcome and restenosis rate in a single-center series of 21 consecutive patients with acute and mid-term results. Clin Neurol Neurosurg. 2013;115(6):741-7.*
75. *Lee JH, Jo SM, Jo KD, et al. Comparison of Drug-eluting Coronary Stents, Bare Coronary Stents and Self-expanding Stents in Angioplasty of Middle Cerebral Artery Stenoses. J Cerebrovasc Endovasc Neurosurg. 2013;15(2):85-95.*
76. *Lu H, Zheng P, Zhang W. Long-term outcome of drug-eluting stenting for stenoses of the intracranial vertebrobasilar artery and vertebral ostium. J Neurointerv Surg. 2013;5(5):435-9.*
77. *Park S, Kim JH, Kwak JK, et al. Intracranial stenting for severe symptomatic stenosis: self-expandable versus balloon-expandable stents. Interv Neuroradiol. 2013; 19(3): 276-82.*
78. *Park S, Lee DG, Chung WJ, et al. Long-term Outcomes of Drug-eluting Stents in Symptomatic Intracranial Stenosis. Neurointervention. 2013;8(1):9-14.*
79. *Rohde S, Seckinger J, Hähnel S, et al. Stent design lowers angiographic but not clinical adverse events in stenting of symptomatic intracranial stenosis - results of a single center study with 100 consecutive patients. Int J Stroke. 2013;8(2):87-94.*
80. *Zhang L, Huang Q, Zhang Y, et al. A single-center study of Wingspan stents for symptomatic atherosclerotic stenosis of the middle cerebral artery. J Clin Neurosci. 2013;20(3):362-6.*
81. *Chang YH, Hwang SK, Kwon OK. Primary angioplasty for symptomatic atherosclerotic middle cerebral artery stenosis. J Cerebrovasc Endovasc Neurosurg. 2014;16(3):166-74.*
82. *Derdeyn CP, Chimowitz MI, Lynn MJ, et al. Aggressive medical treatment with or without stenting in high-risk patients with intracranial artery stenosis (SAMMPRIS): the final results of a randomised trial. Lancet. 2014;383(9914):333-41.*
83. *Silber T, Ziemann U, Ernemann U, et al. Analysis of periinterventional complications of intracranial angioplasty and stenting: a single center experience. Eur J Radiol. 2014;83(12):2190-2195.*
84. *Yu SC, Leung TW, Lee KT, et al. Angioplasty and stenting of intracranial atherosclerosis with the Wingspan system: 1-year clinical and radiological outcome in a single Asian center. J Neurointerv Surg. 2014;6(2):96-102.*
85. *Miao Z, Song L, Liebeskind DS, et al. Outcomes of tailored angioplasty and/or stenting for symptomatic intracranial atherosclerosis: a prospective cohort study after SAMMPRIS. J Neurointerv Surg. 2015;7(5):331-5.*
86. *Okada H, Terada T, Tanaka Y, et al. Reappraisal of primary balloon angioplasty without stenting for patients with symptomatic middle cerebral artery stenosis. Neurol Med Chir (Tokyo). 2015;55(2):133-40.*
87. *Feng Z, Duan G, Zhang P, et al. Enterprise stent for the treatment of symptomatic intracranial atherosclerotic stenosis: an initial experience of 44 patients. BMC Neurol. 2015;15:187.*
88. *Li TX, Gao BL, Cai DY, et al. Wingspan Stenting for Severe Symptomatic Intracranial Atherosclerotic Stenosis in 433 Patients Treated at a Single Medical Center. PLoS One. 2015;10(9):e0139377.*
89. *Miao Z, Zhang Y, Shuai J, et al. Thirty-Day Outcome of a Multicenter Registry Study of Stenting for Symptomatic Intracranial Artery Stenosis in China. Stroke. 2015; 46(10):2822-9.*
90. *von Schoenfeldt P, Krützelmann A, Bußmeyer M, et al. Elective treatment of intracranial stenosis with the balloon-expandable Pharos Vitesse stent: 30-day stroke rate and complications. J Neurointerv Surg. 2015;7(3):188-93.*
91. *Wang ZL, Gao BL, Li TX, et al. Symptomatic intracranial vertebral artery atherosclerotic stenosis (≥70%) with concurrent contralateral vertebral atherosclerotic diseases in 88 patients treated with the intracranial stenting. Eur J Radiol. 2015;84(9):1801-4.*
92. *Xiong Y, Zhou Z, Lin H, et al. The safety and long-term outcomes of angioplasty and stenting in symptomatic intracranial atherosclerotic stenosis. Int J Cardiol. 2015; 179:23-4.*
93. *Yin R, Chang F, Hong B, et al. Safety and Efficacy Analyses of Angioplasty and Stenting for Severe Intracranial Arterial Stenosis: A Single-Center Retrospective Study in China. Med Sci Monit. 2015;21:3311-9.*
94. *Zaidat OO, Fitzsimmons BF, Woodward BK, et al. Effect of a balloon-expandable intracranial stent vs medical therapy on risk of stroke in patients with symptomatic intracranial stenosis: the VISSIT randomized clinical trial. JAMA. 2015;313(12): 1240-8.*
95. *Al Said Y, Kurdi K, Baeesa SS, et al. Outcome of intracranial arterial stenting of symptomatic atherosclerotic disease: A single center experience from Saudi Arabia. Neurosciences (Riyadh). 2016;21(4):366-371.*
96. *Bai WX, Gao BL, Li TX, et al. Wingspan stenting can effectively prevent long-term strokes for patients with severe symptomatic atherosclerotic basilar stenosis. Interv Neuroradiol. 2016;22(3):318-24.*
97. *Cheng L, Jiao L, Gao P, et al. Risk factors associated with in-hospital serious adverse events after stenting of severe symptomatic intracranial stenosis. Clin Neurol Neurosurg. 2016;147:59-63.*
98. *Duan G, Feng Z, Zhang L, et al. Solitaire stents for the treatment of complex symptomatic intracranial stenosis after antithrombotic failure: safety and efficacy evaluation. J Neurointerv Surg. 2016;8(7):680-4.*
99. *Gao P, Wang D, Zhao Z, et al. Multicenter Prospective Trial of Stent Placement in Patients with Symptomatic High-Grade Intracranial Stenosis. AJNR Am J Neuroradiol. 2016;37(7):1275-80.*
100. *Liu L, Zhao X, Mo D, et al. Stenting for symptomatic intracranial vertebrobasilar artery stenosis: 30-day results in a high-volume stroke center. Clin Neurol Neurosurg. 2016;143:132-8.*
101. *Wang X, Wang Z, Wang C, et al. Application of the Enterprise Stent in Atherosclerotic Intracranial Arterial Stenosis: A Series of 60 Cases. Turk Neurosurg. 2016;26(1):69-76.*
102. *Wang ZL, Gao BL, Li TX, et al. Severe symptomatic intracranial internal carotid artery stenosis treated with intracranial stenting: a single center study with 58 patients. Diagn Interv Radiol. 2016;22(2):178-83.*
103. *Wang ZL, Gao BL, Li TX, et al. Outcomes of middle cerebral artery angioplasty and stenting with Wingspan at a high-volume center. Neuroradiology. 2016;58(2): 161-9.*
104. *Zhao T, Zhu WY, Xiong XY, et al. Safety and Efficacy of Wingspan Stenting for Severe Symptomatic Atherosclerotic Stenosis of the Middle Cerebral Artery: Analysis of 278 Continuous Cases. J Stroke Cerebrovasc Dis. 2016;25(10):2368-72.*
105. *Zhang F, Liu L. Complication of Stenting in Intracranial Arterial Stenosis. Arch Iran Med. 2016;19(5):317-22.*
106. *Markus HS, Larsson SC, Kuker W, et al. Stenting for symptomatic vertebral artery stenosis: The Vertebral Artery Ischaemia Stenting Trial. Neurology. 2017; 89(12):1229-1236.*
107. *Yeo LL, Wu YM, Chen YL, et al. MRI audit of complications in intracranial stenosis treated with Wingspan device. J Neurointerv Surg. 2017;9(5):466-470.*
108. *Ueda T, Takada T, Nogoshi S, et al. Long-Term Outcome of Balloon Angioplasty Without Stenting for Symptomatic Middle Cerebral Artery Stenosis. J Stroke Cerebrovasc Dis. 2018;27(7):1870-1877.*
109. *Gruber P, Garcia-Esperon C, Berberat J, et al. Neuro Elutax SV drug-eluting balloon versus Wingspan stent system in symptomatic intracranial high-grade stenosis: a single-center experience. J Neurointerv Surg. 2018;10(12):e32.*
110. *Maier IL, Karch A, Lipke C, et al. Transluminal angioplasty and stenting versus conservative treatment in patients with symptomatic basilar artery stenosis : Perspective for future clinical trials. Clin Neuroradiol. 2018;28(1):33-38.*
111. *Yu SCH, Lau TWW, Wong SSM, et al. Long-Term Evolutionary Change in the Lumen of Intracranial Atherosclerotic Stenosis Following Angioplasty and Stenting. Oper Neurosurg (Hagerstown). 2018;14(2):128-138.*
112. *Zhao J, Li X, Chi LX, et al. Concomitant Asymptomatic Intracranial Atherosclerotic Stenosis Increase the 30-Day Risk of Stroke in Patients Undergoing Symptomatic Intracranial Atherosclerotic Stenosis Stenting. J Stroke Cerebrovasc Dis. 2018;27(2):479-485.*
113. *Wang Y, Ma Y, Gao P, et al. Primary Angioplasty without Stenting for Symptomatic, High-Grade Intracranial Stenosis with Poor Circulation. AJNR Am J Neuroradiol. 2018;39(8):1487-1492.*
114. *Baik SH, Kwak HS, Chung GH, et al. Balloon-expandable stents for treatment of symptomatic middle cerebral artery stenosis: Clinical outcomes during long-term follow-up. Interv Neuroradiol. 2018;24(6):666-673.*
115. *Peng G, Zhang J, Jia B, et al. Submaximal primary angioplasty for symptomatic intracranial atherosclerosis: peri-procedural complications and long-term outcomes. Neuroradiology. 2019;61(1):97-102.*
116. *Zhou Y, Wang L, Zhang JR, et al. Angioplasty and stenting for severe symptomatic atherosclerotic stenosis of intracranial vertebrobasilar artery. J Clin Neurosci. 2019;63:17-21.*
117. *Salik AE, Selcuk HH, Zalov H, et al. Medium-term results of undersized angioplasty and stenting for symptomatic high-grade intracranial atherosclerotic stenosis with Enterprise. Interv Neuroradiol. 2019;25(5):484-490.*
118. *Zhang Y, Rajah GB, Liu P, et al. Balloon-mounted versus self-expanding stents for symptomatic intracranial vertebrobasilar artery stenosis combined with poor collaterals. Neurol Res. 2019;41(8):704-713.*
119. *Gross BA, Desai SM, Walker G, et al. Balloon-mounted stents for acute intracranial large vessel occlusion secondary to presumed atherosclerotic disease: evolution in an era of supple intermediate catheters. J Neurointerv Surg. 2019;11(10): 975-978.*
120. *Park SC, Cho SH, Kim MK, et al. Long-term Outcome of Angioplasty Using a Wingspan Stent, Post-Stent Balloon Dilation and Aggressive Restenosis Management for Intracranial Arterial Stenosis. Clin Neuroradiol. 2020;30(1): 159-169.*
121. *Zhang Y, Sun Y, Li X, et al. Early versus delayed stenting for intracranial atherosclerotic artery stenosis with ischemic stroke. J Neurointerv Surg. 2020;12(3): 274-278.*
122. ***Summary results***

*
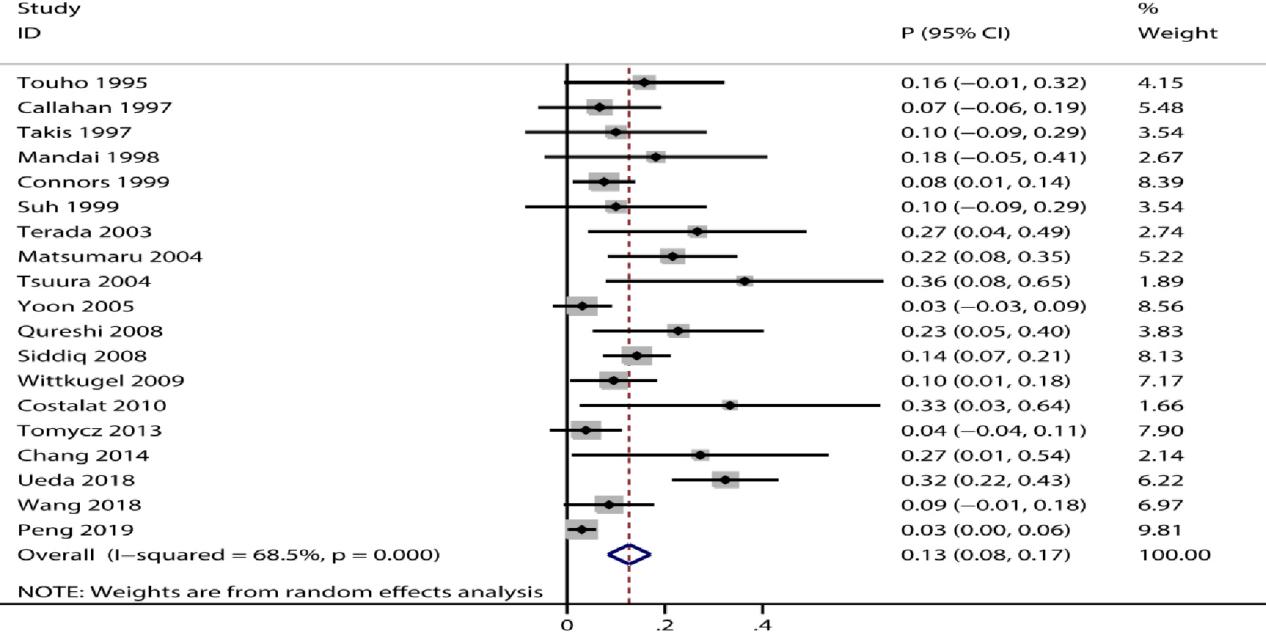
*

*Figure S1. Pooled incidence of restenosis after balloon angioplasty*

*
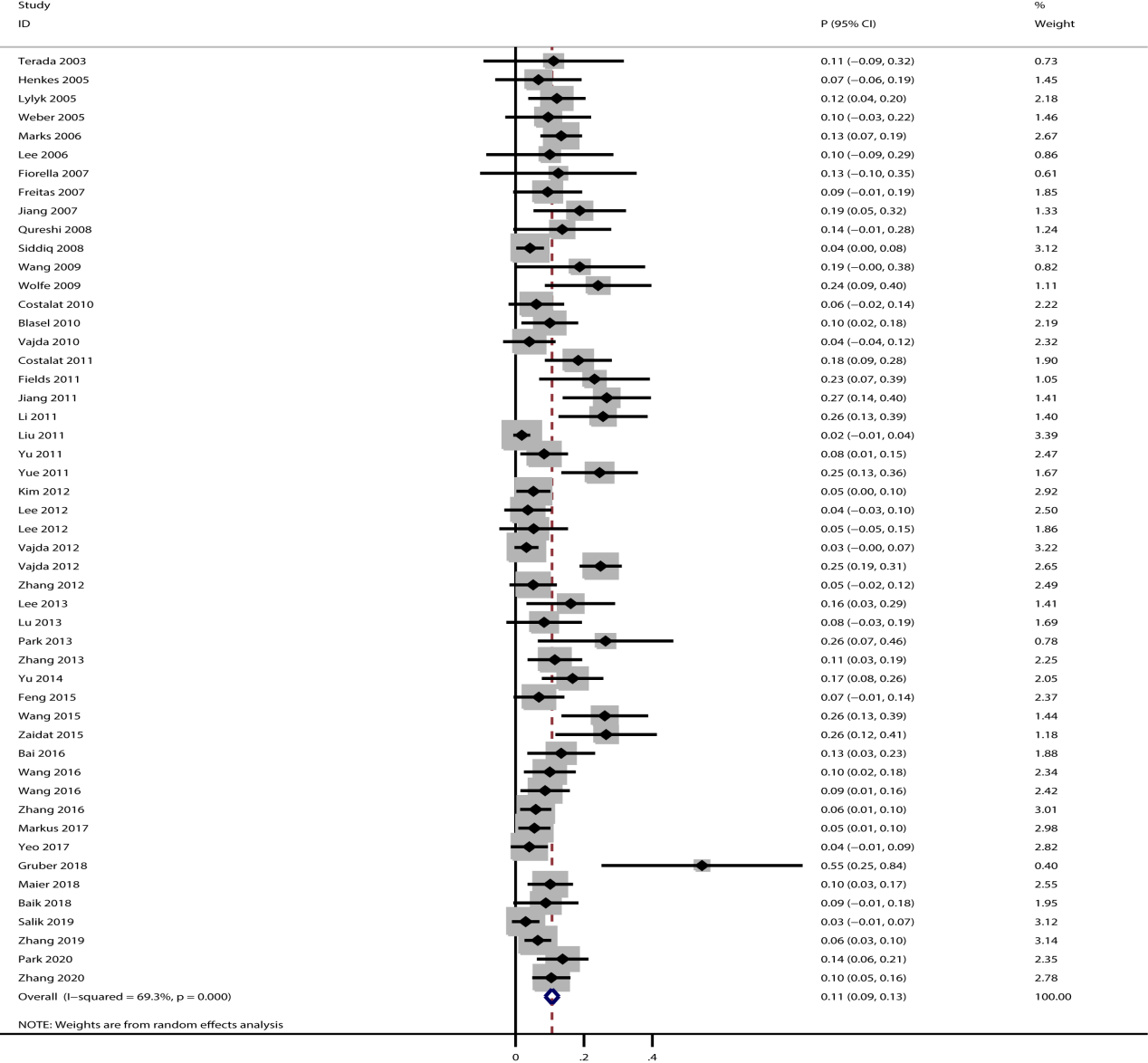
*

*Figure S2. Pooled incidence of restenosis after stenting*

*
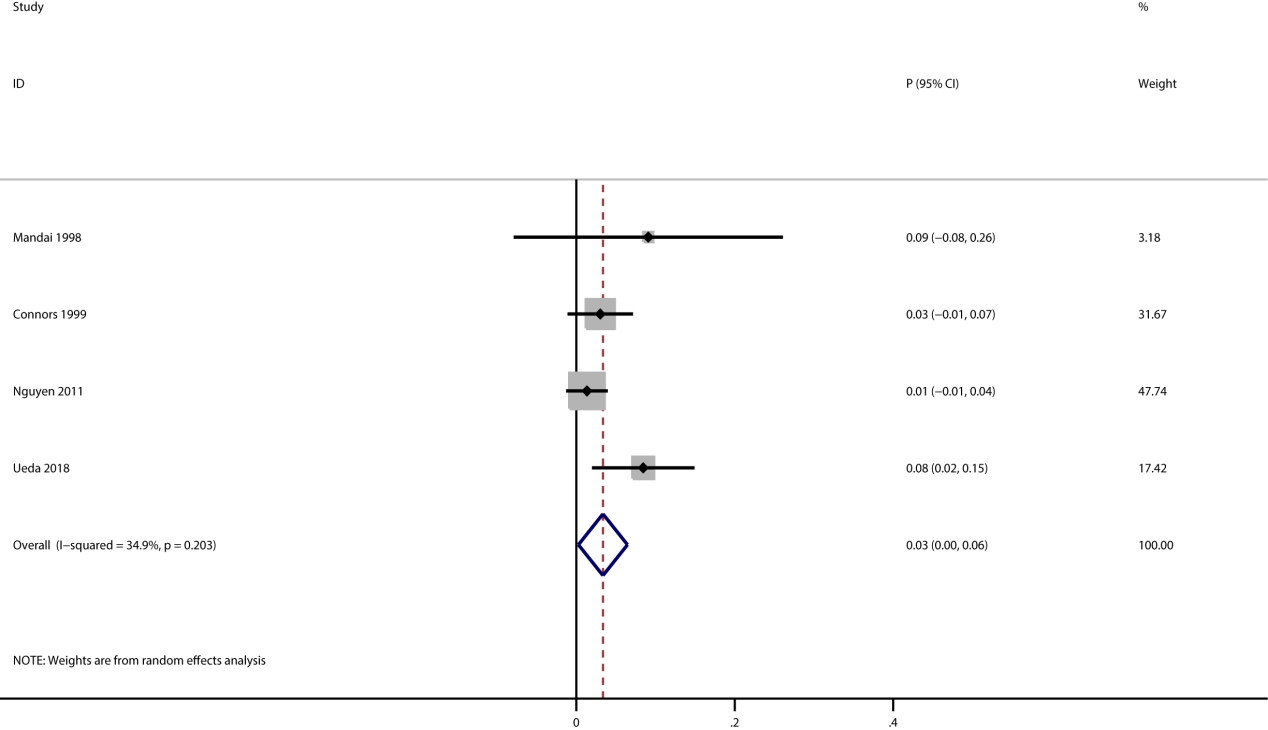
*

*Figure S3. Pooled incidence of transient ischemic attack after balloon angioplasty*

*
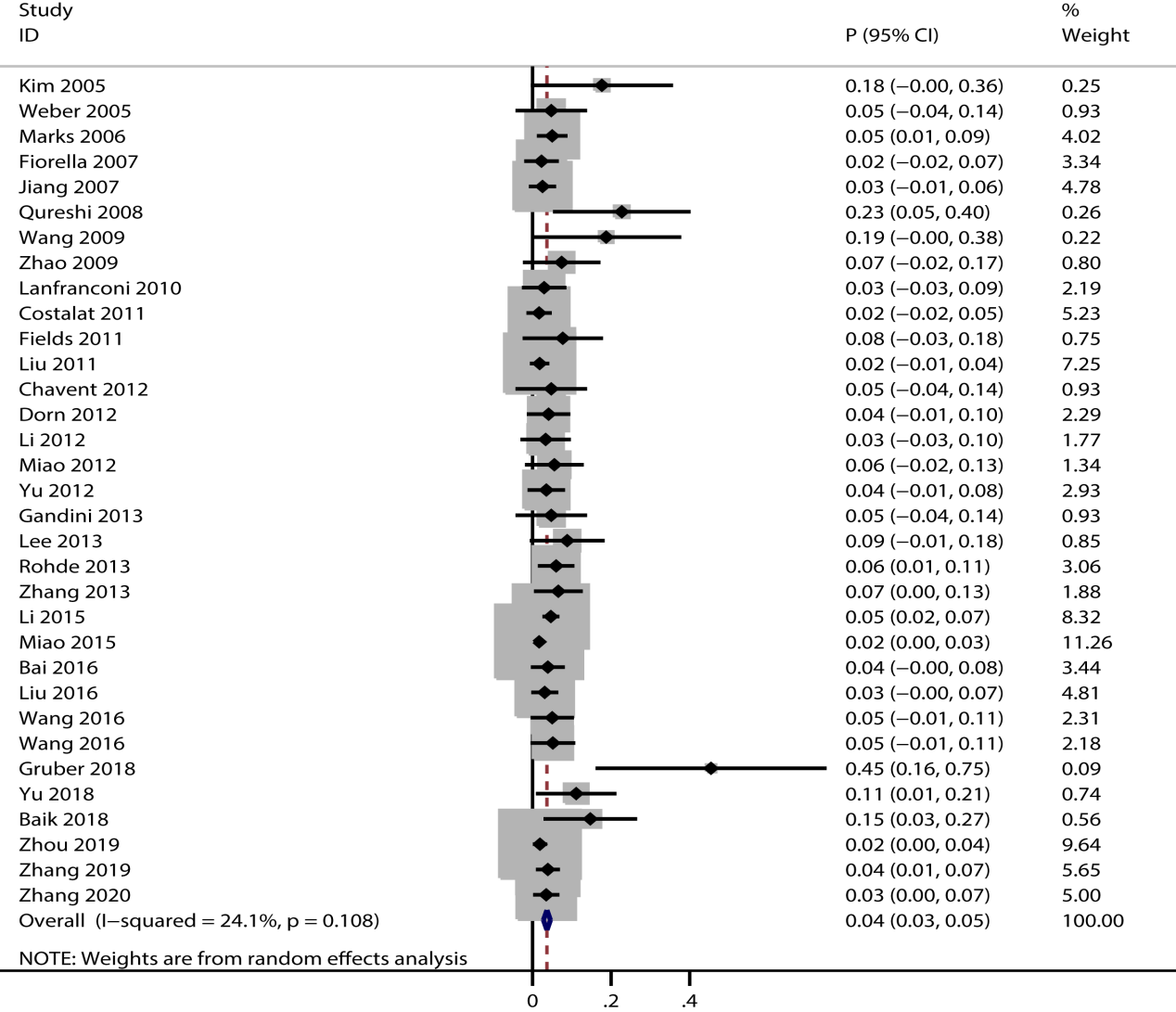
*

*Figure S4. Pooled incidence of transient ischemic attack after stenting*

*
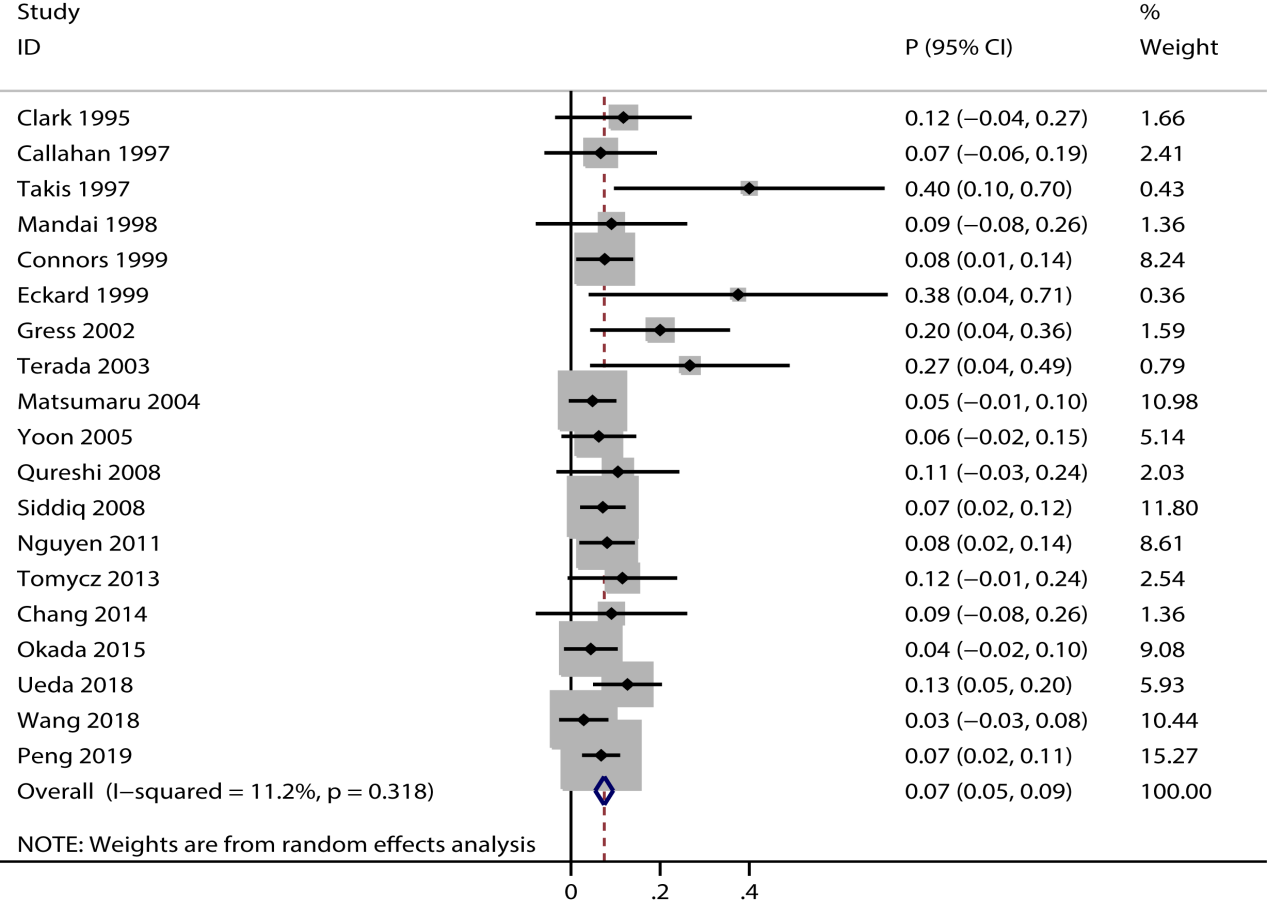
*

*Figure S5. Pooled incidence of stroke after balloon angioplasty*

*
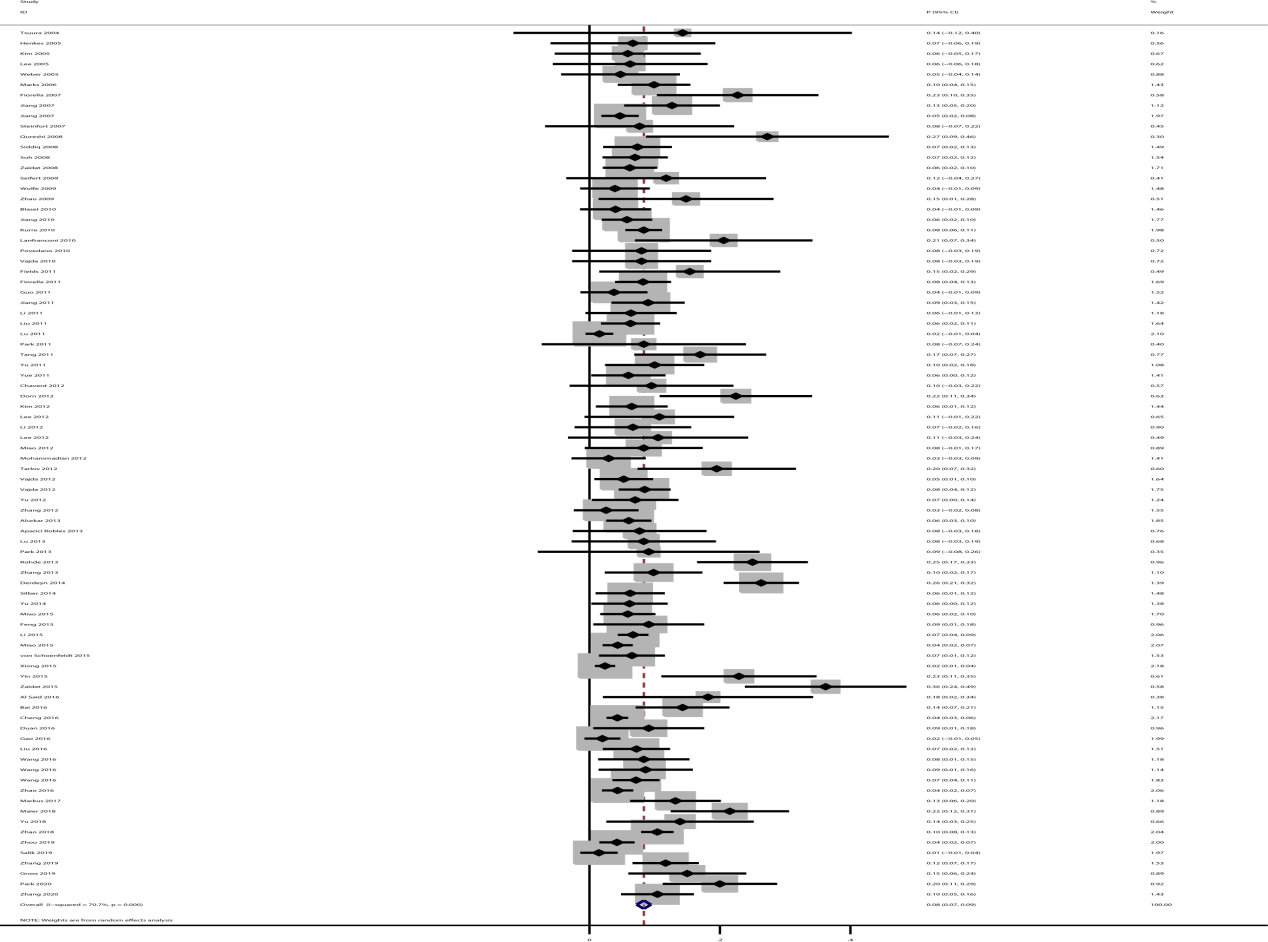
*

*Figure S6. Pooled incidence of stroke after stenting*

***
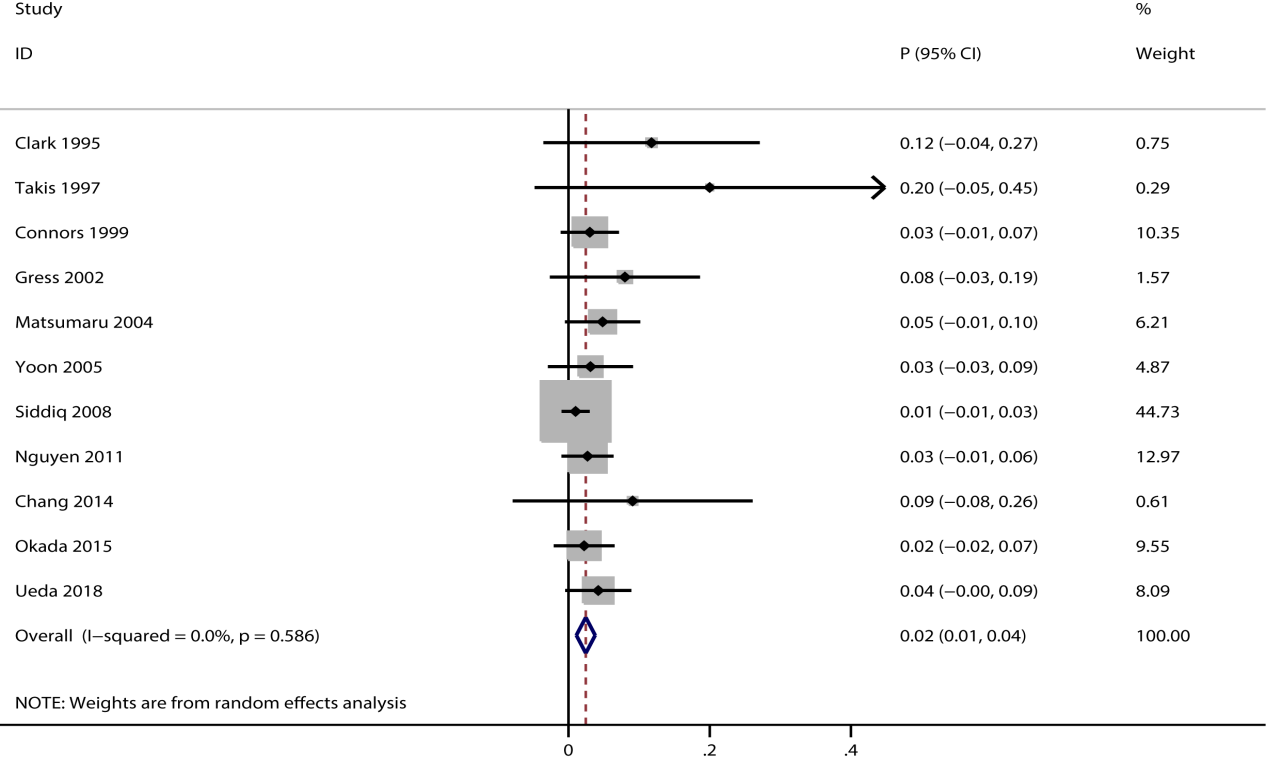
***

*Figure S7. Pooled incidence of death after balloon angioplasty*

*
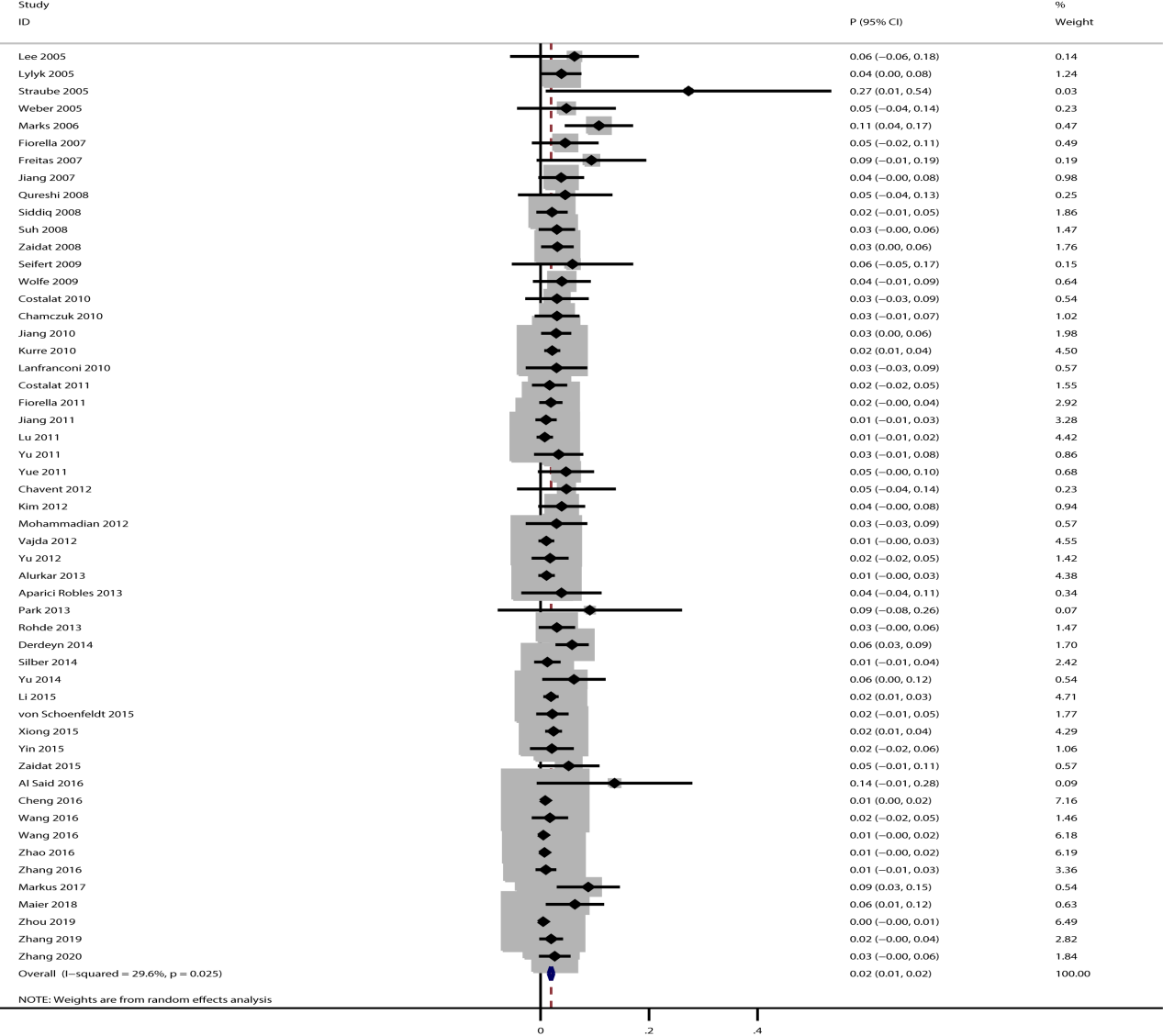
*

*Figure S8. Pooled incidence of death after stenting*

*
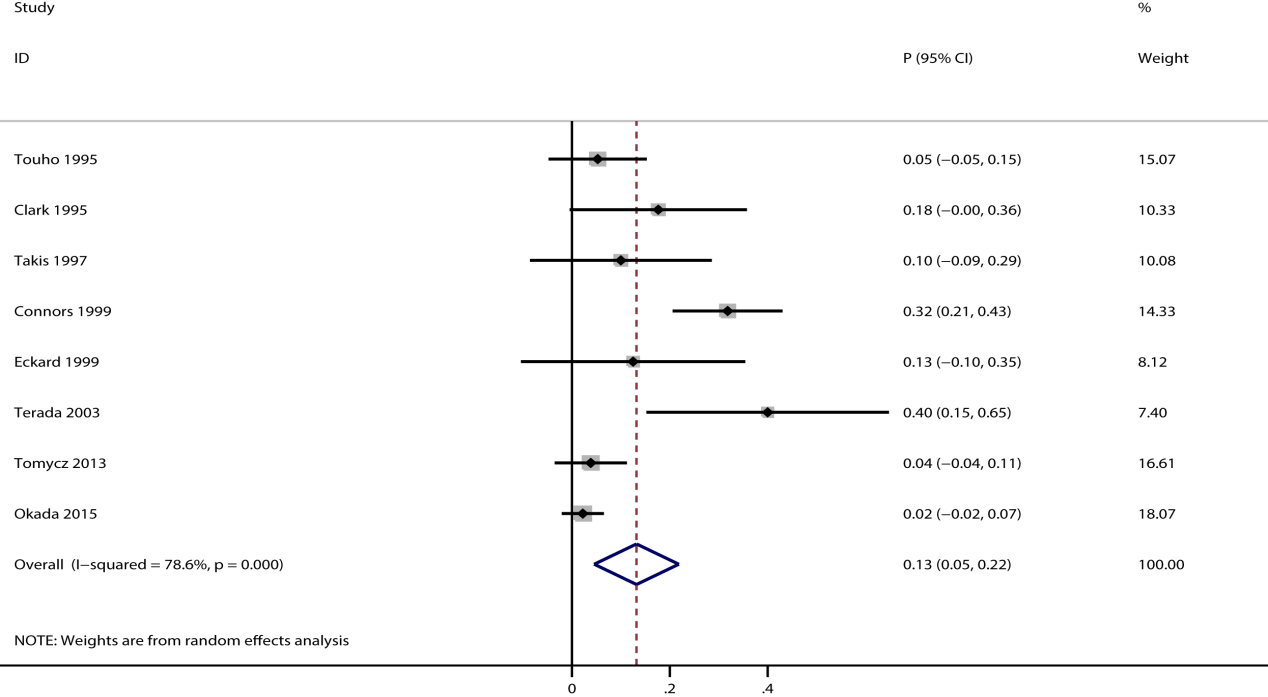
*

*Figure S9. Pooled incidence of dissection after balloon angioplasty*

*
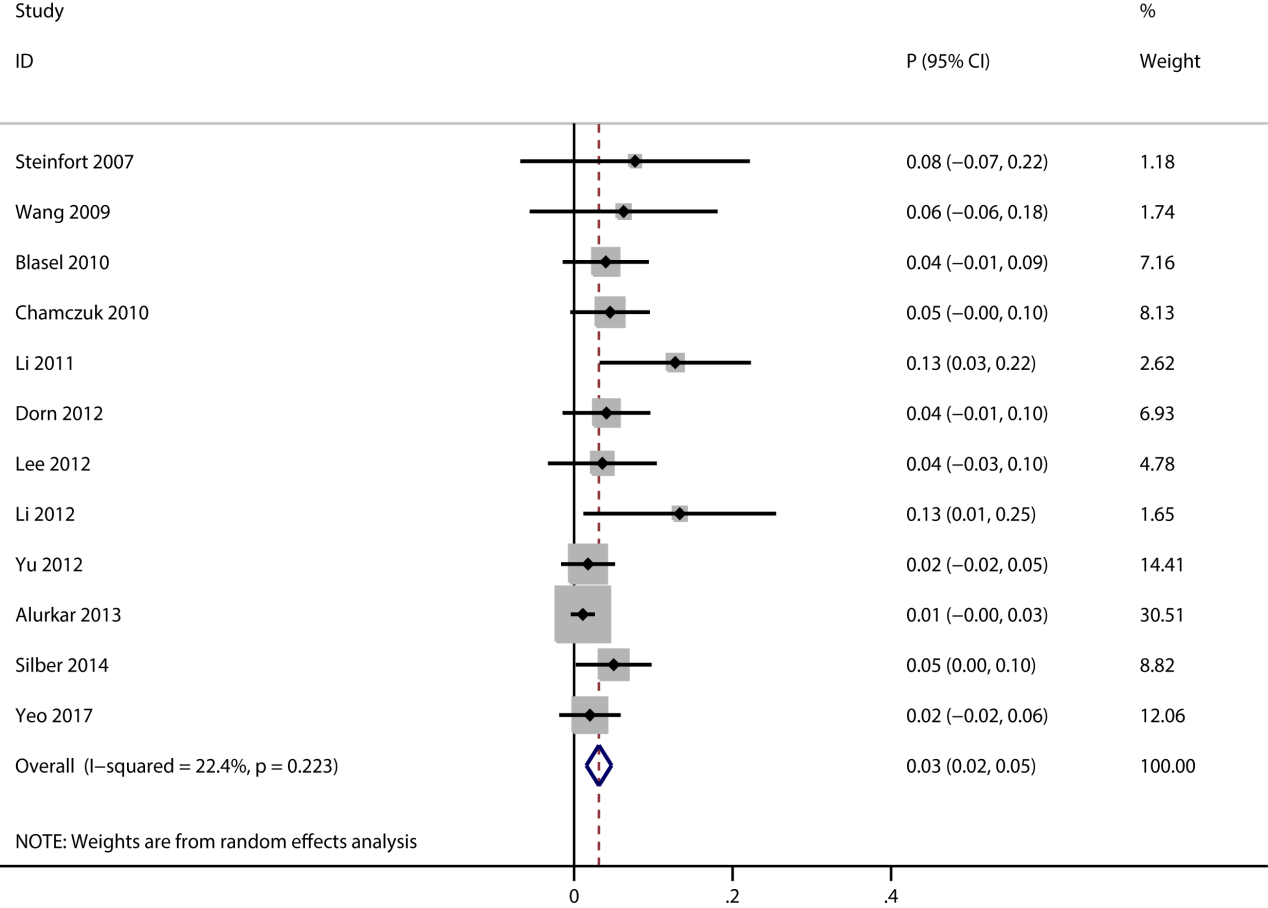
*

*Figure S10. Pooled incidence of dissection after stenting*

1. ***Sensitivity analysis***

*
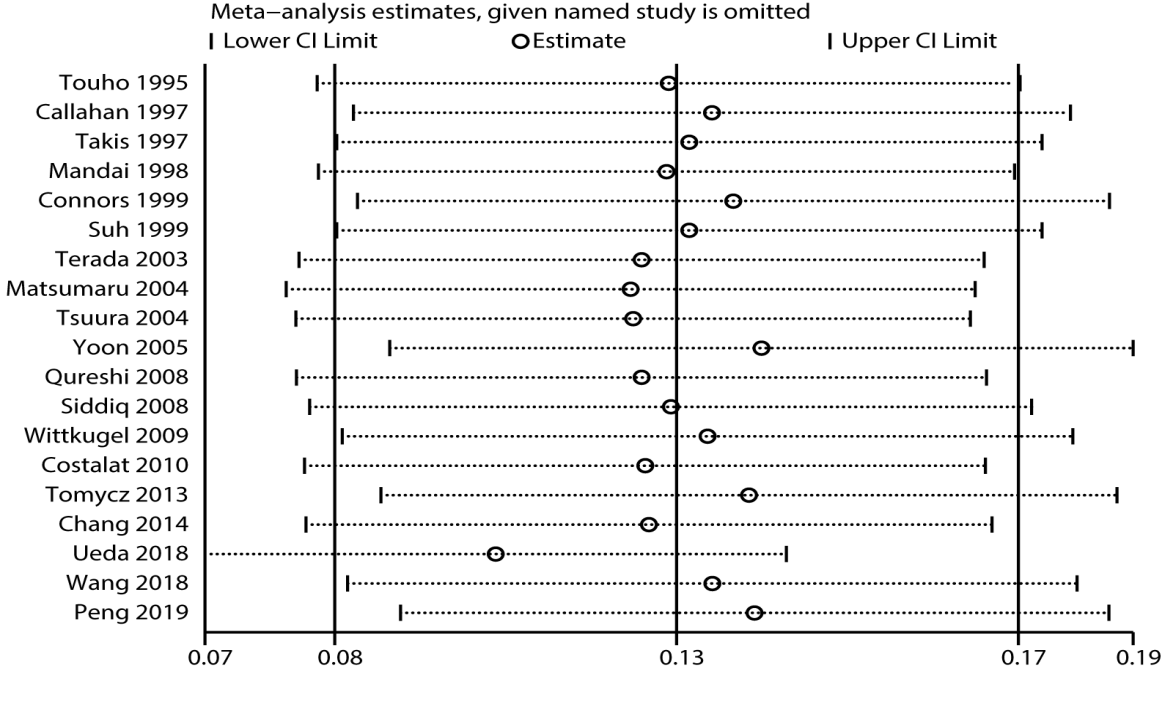
*

*Figure S1. Sensitivity analysis for the pooled incidence of restenosis after balloon angioplasty (10.4%-13.8%)*

*
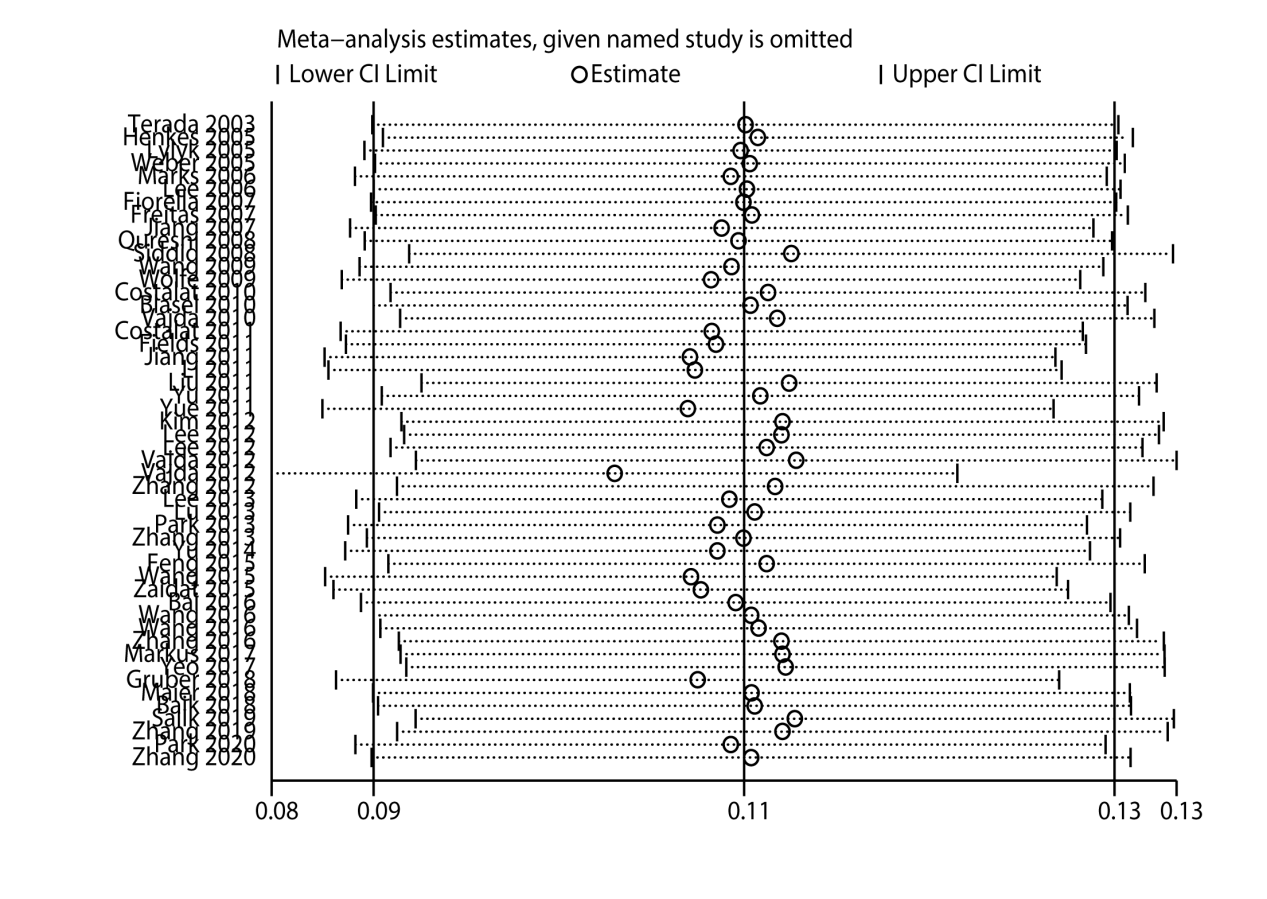
*

*Figure S2. Sensitivity analysis for the pooled incidence of restenosis after stenting (10.0%-10.9%)*

*
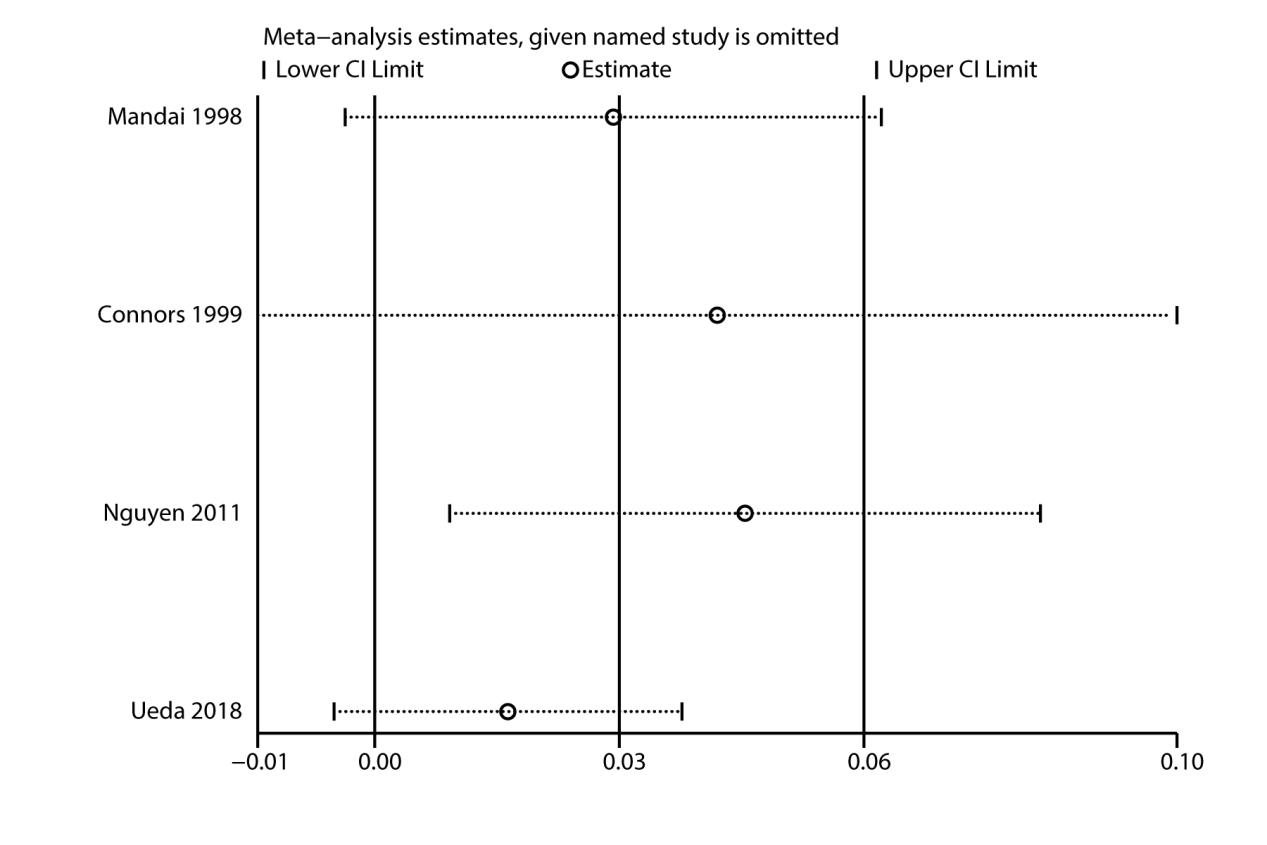
*

*Figure S3. Sensitivity analysis for the pooled incidence of transient ischemic attack after balloon angioplasty (2.0%-5.0%)*

*
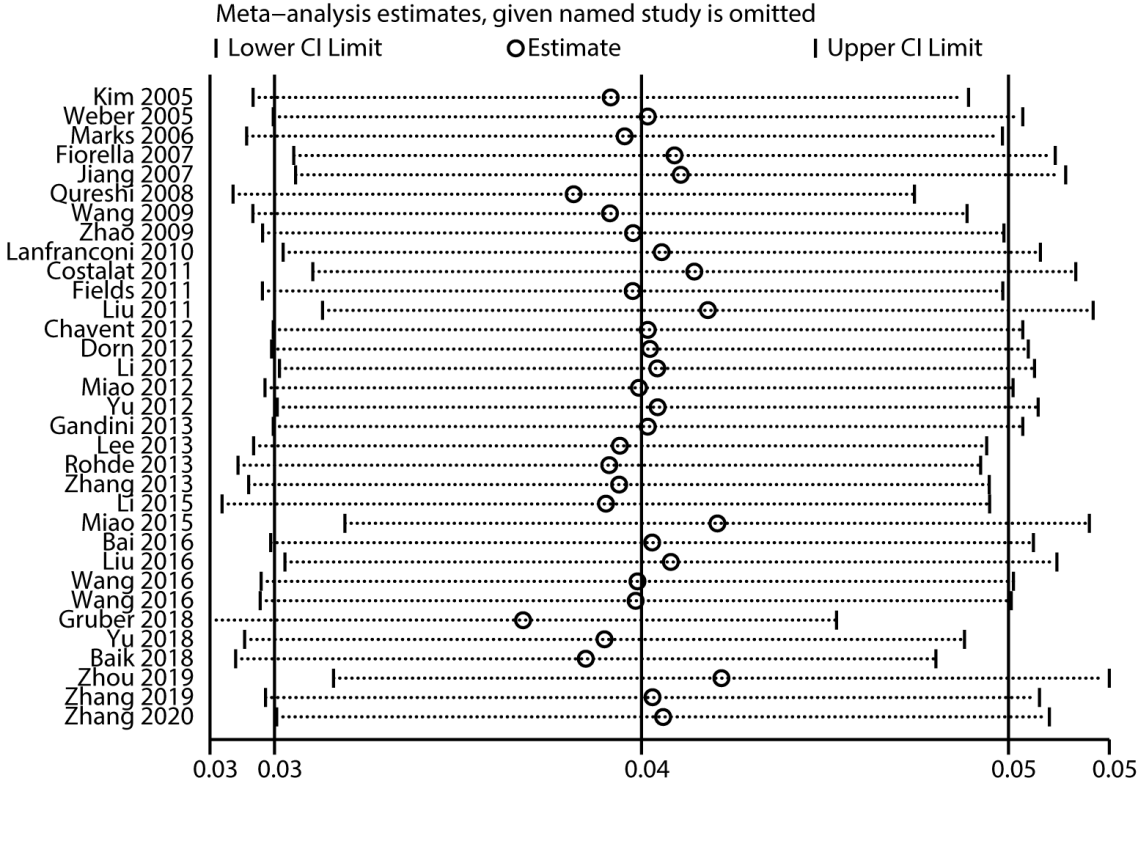
*

*Figure S4. Sensitivity analysis for the pooled incidence of transient ischemic attack after stenting (3.4%-3.9%)*

*
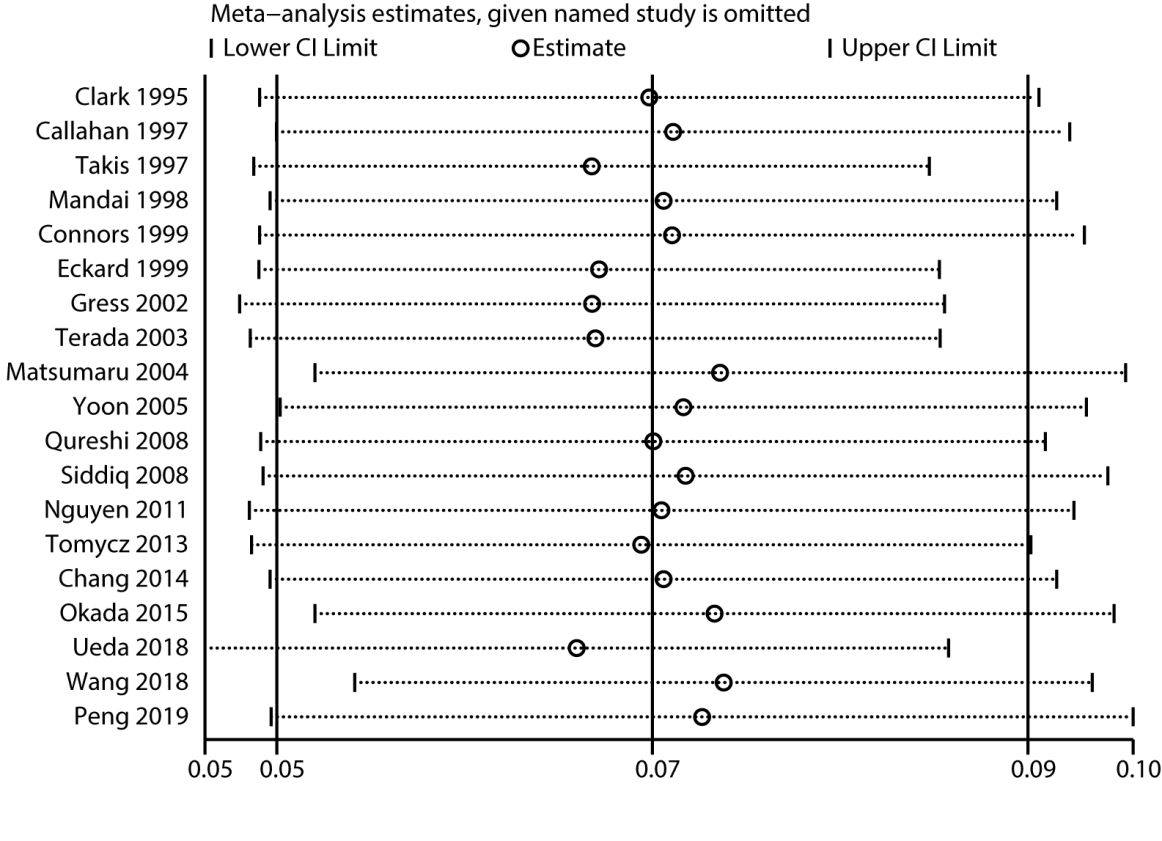
*

*Figure S5. Sensitivity analysis for the pooled incidence of stroke after balloon angioplasty (7.0%-7.8%)*

*
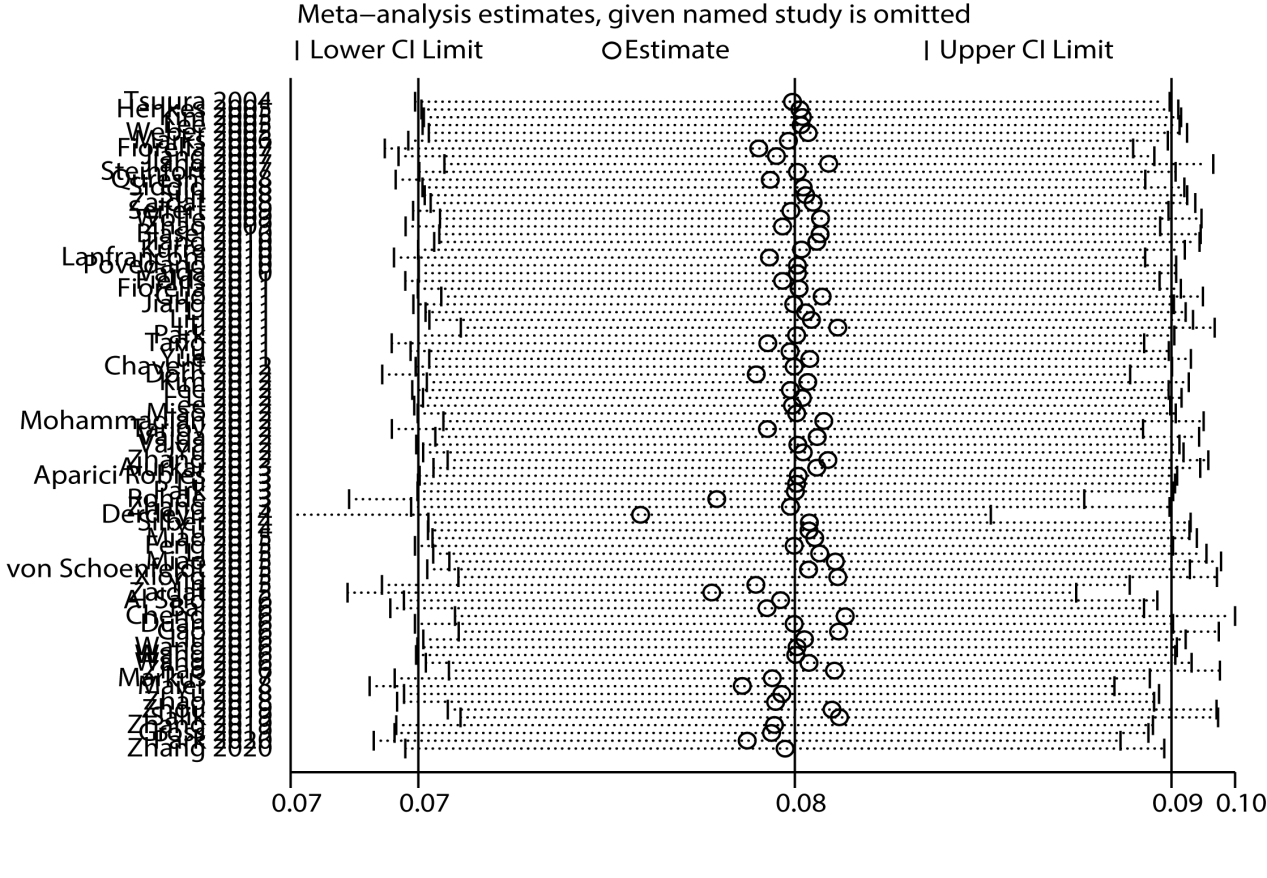
*

*Figure S6. Sensitivity analysis for the pooled incidence of stroke after stenting (7.9%-8.5%)*

*
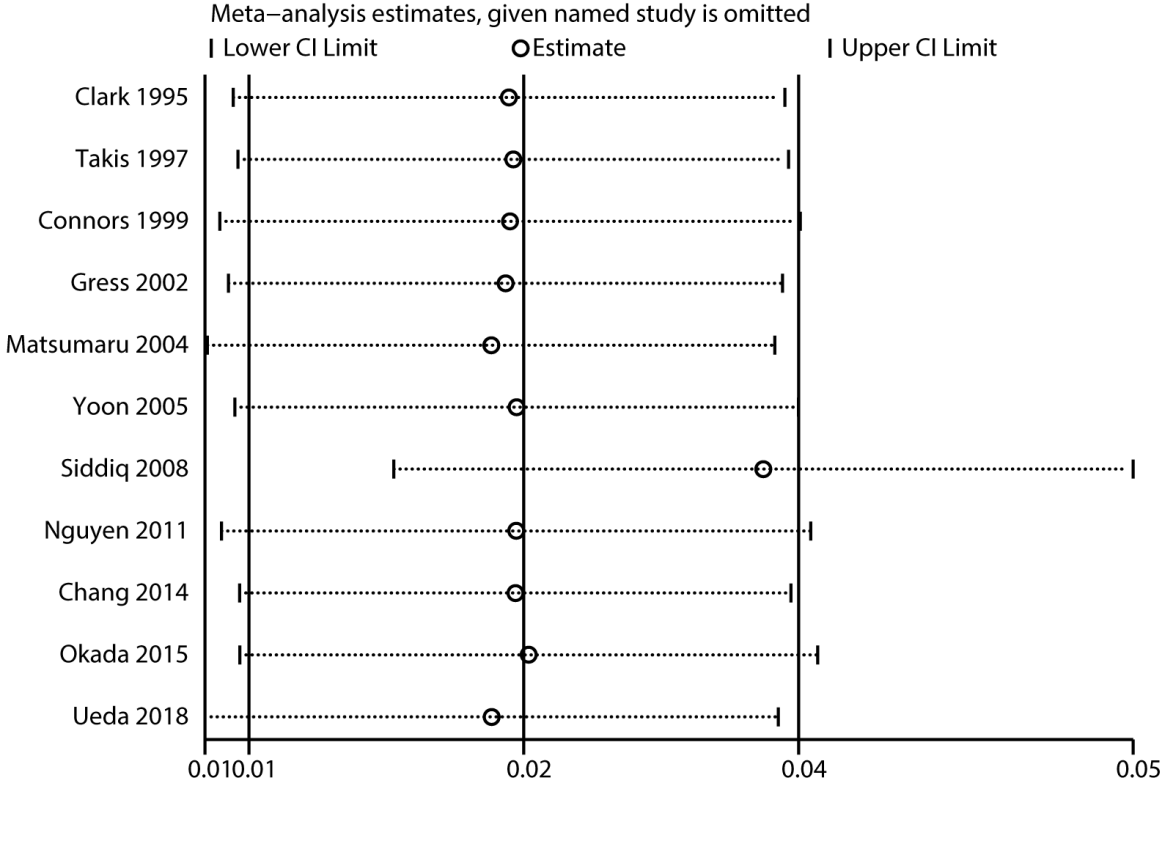
*

*Figure S7. Sensitivity analysis for the pooled incidence of death after balloon angioplasty (2.3%-3.6%)*

*
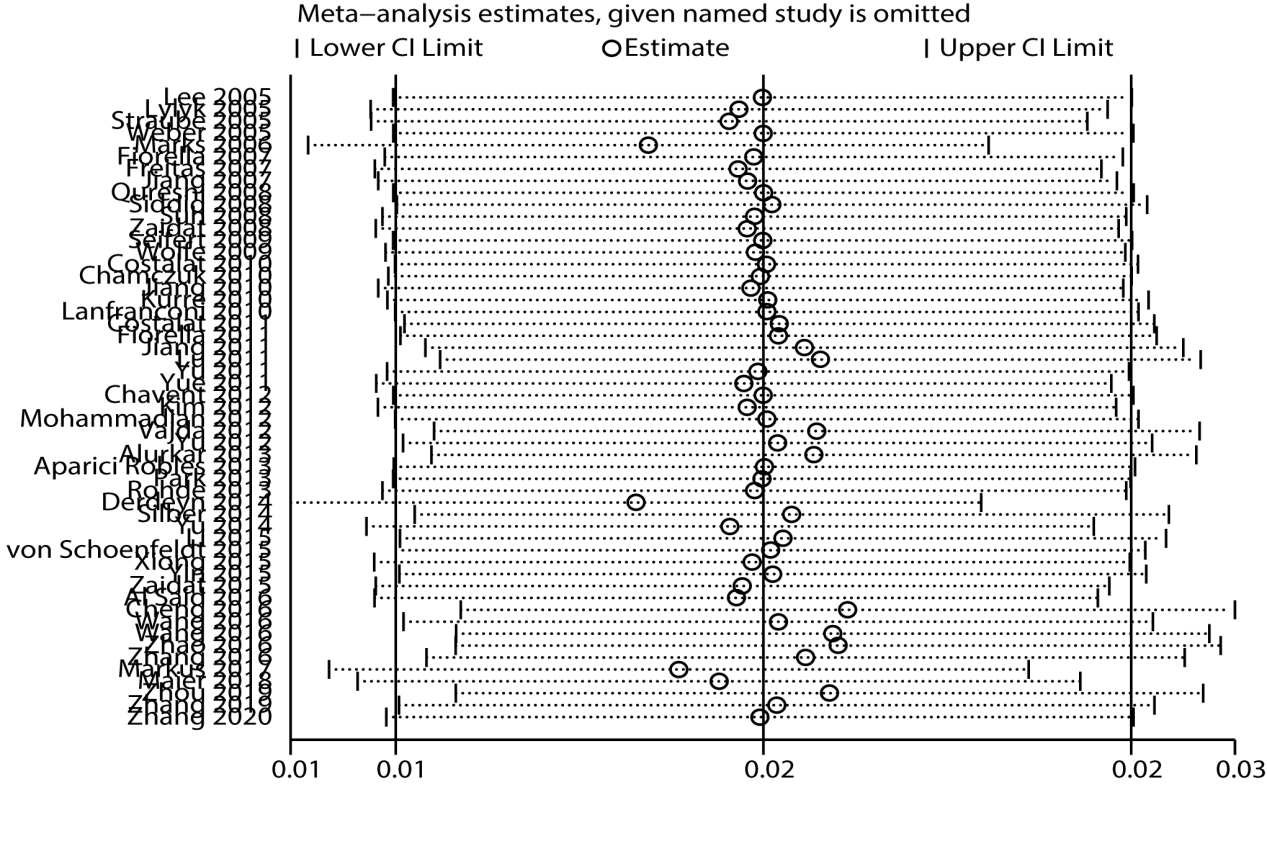
*

*Figure S8. Sensitivity analysis for the pooled incidence of death after stenting (1.8%-2.0%)*

*
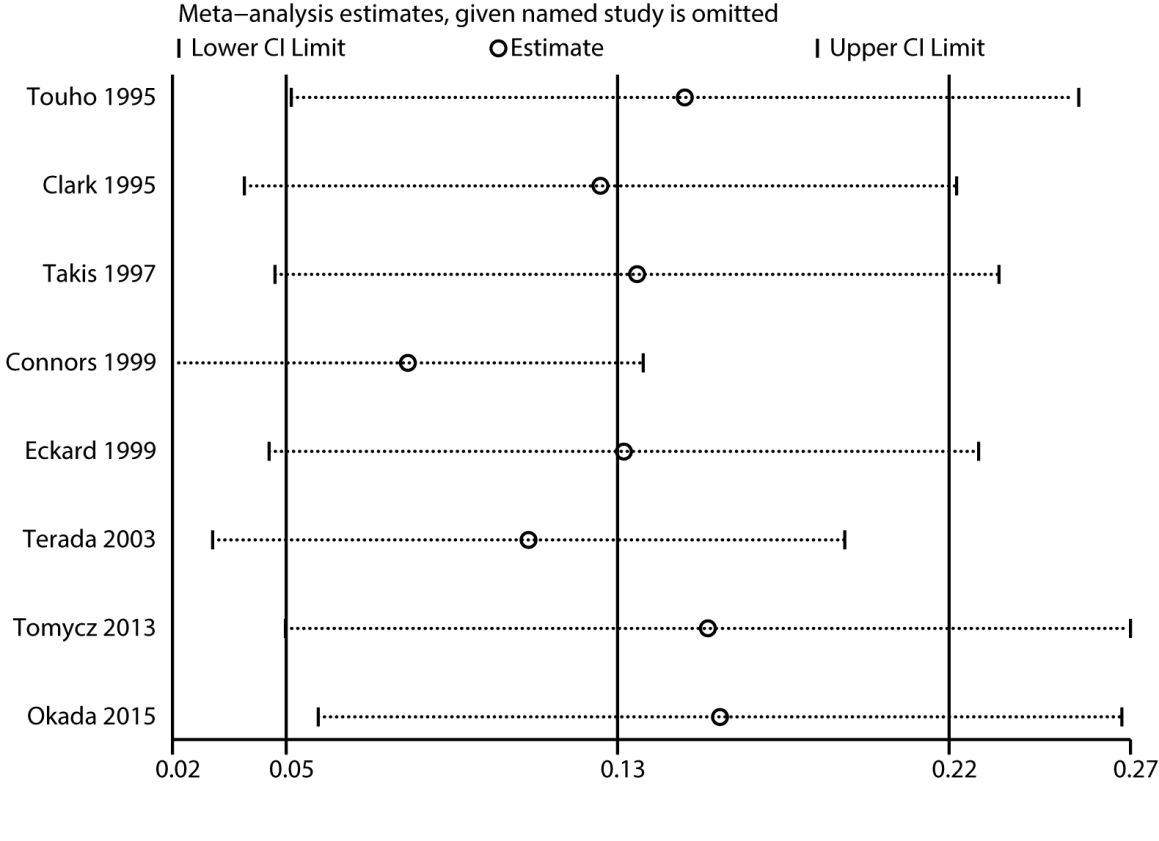
*

*Figure S9. Sensitivity analysis for the pooled incidence of dissection after balloon angioplasty (7.7%-15.9%)*

*
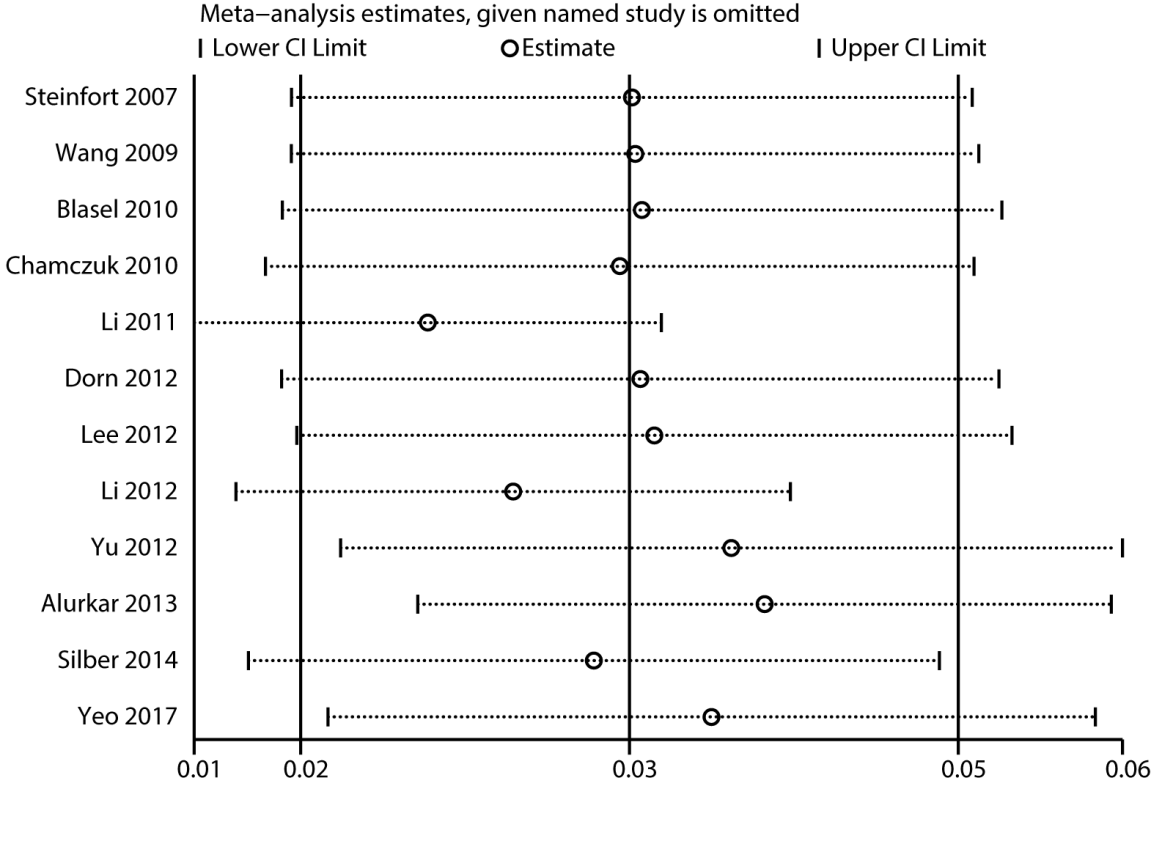
*

*Figure S10. Sensitivity analysis for the pooled incidence of dissection after stenting (2.2%-3.8%)*

1. ***Publication bias***

*
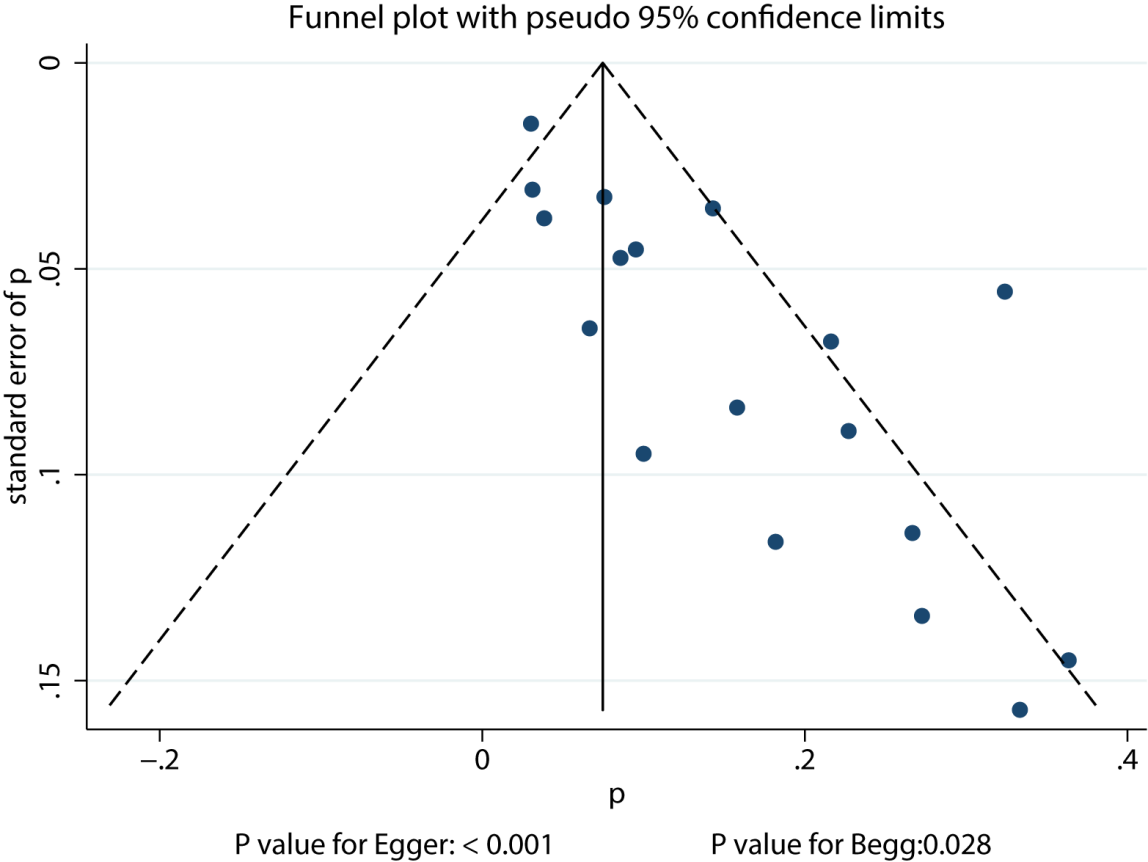
*

*Figure S1. Publication bias test for the pooled incidence of restenosis after balloon angioplasty*

*
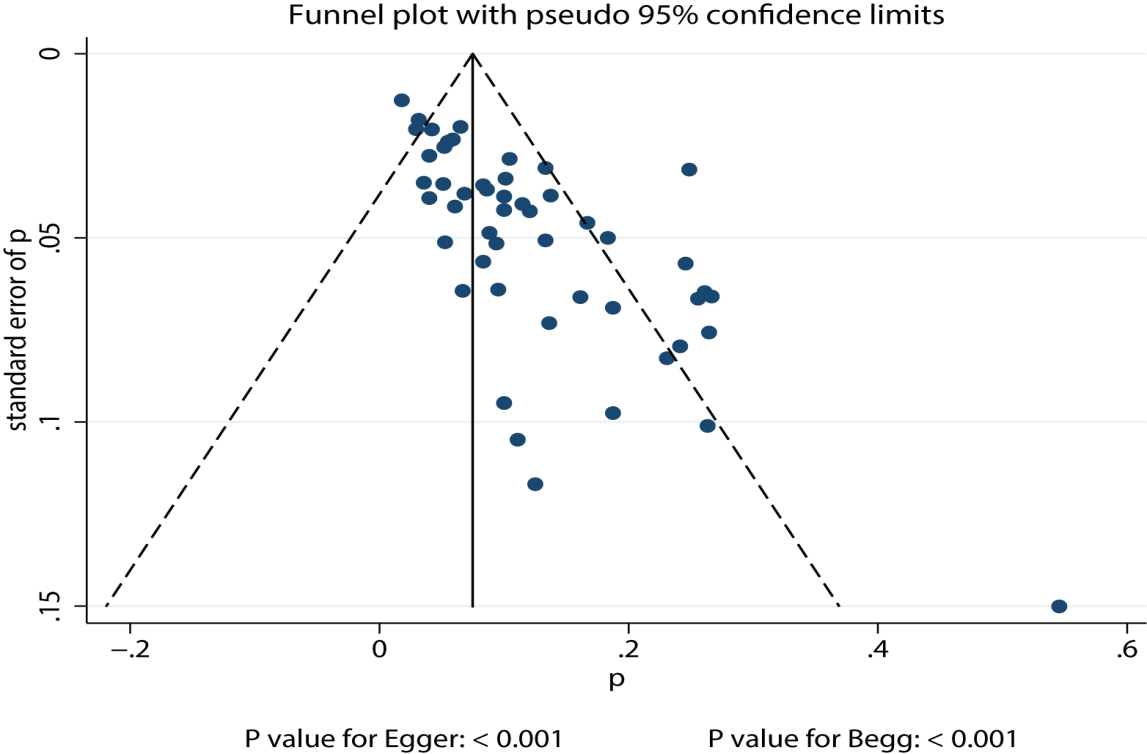
*

*Figure S2. Publication bias test for the pooled incidence of restenosis after stenting*

*
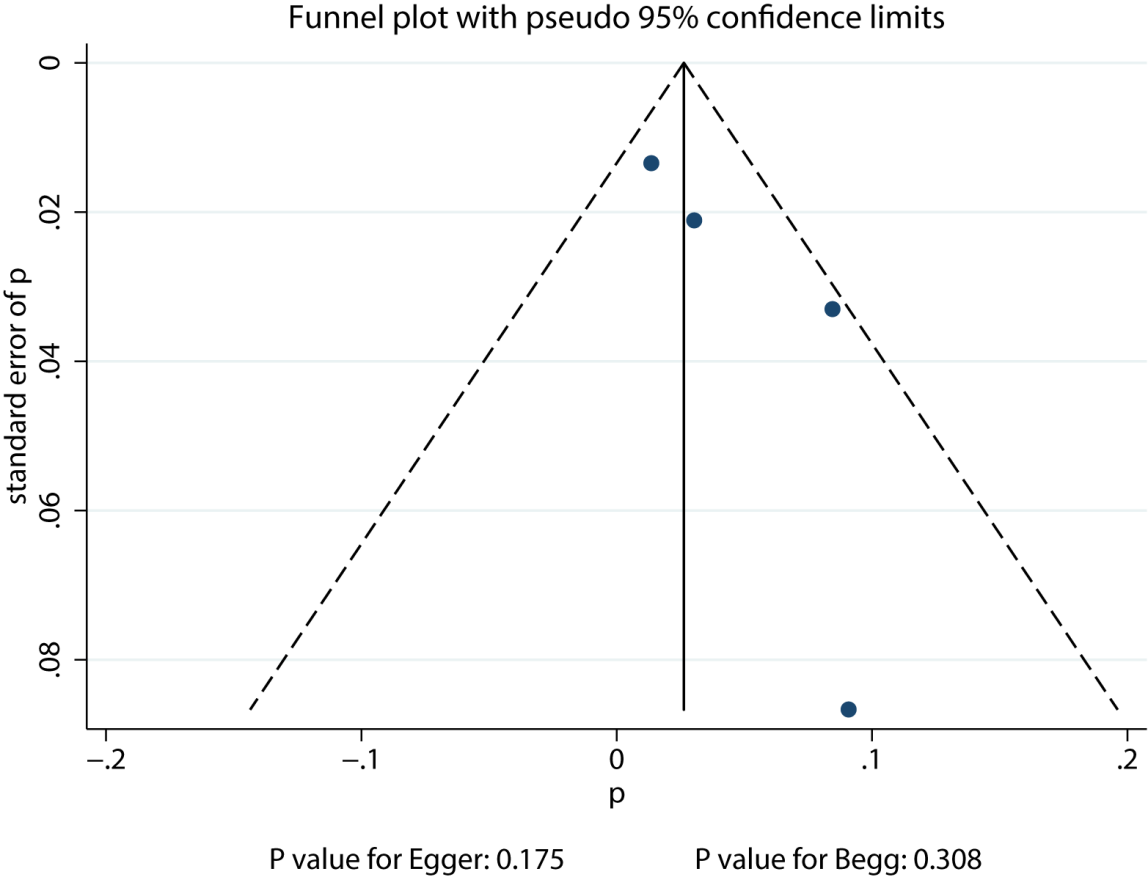
*

*Figure S3. Publication bias test for the pooled incidence of transient ischemic attack after balloon angioplasty*

*
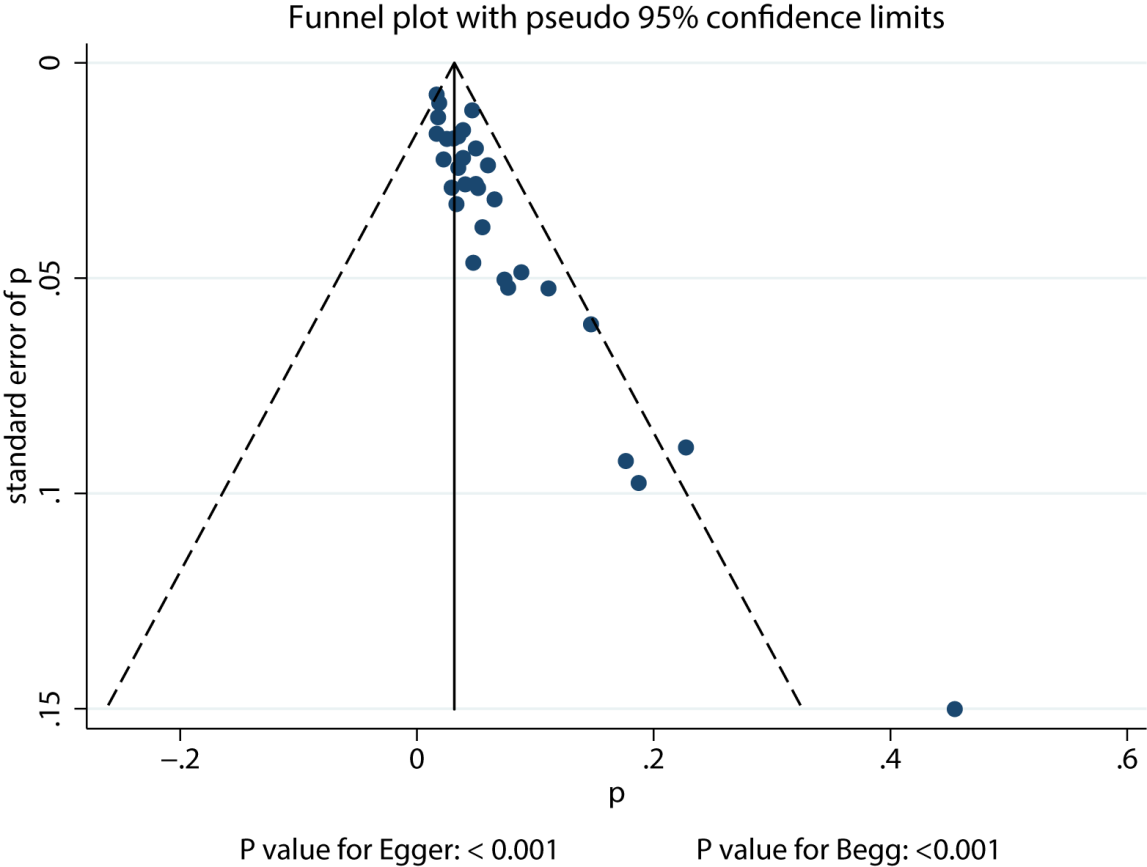
*

*Figure S4. Publication bias test for the pooled incidence of transient ischemic attack after stenting*

*
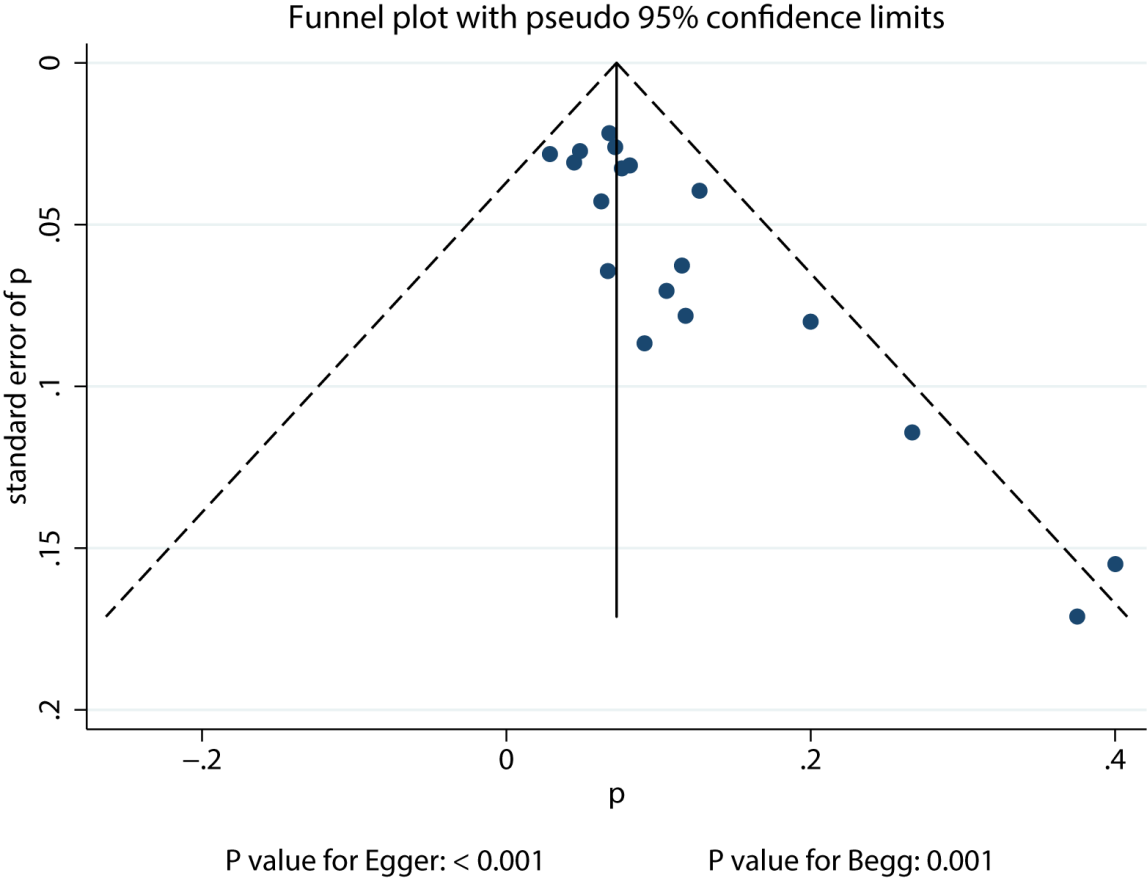
*

*Figure S5. Publication bias test for the pooled incidence of stroke after balloon angioplasty*

*
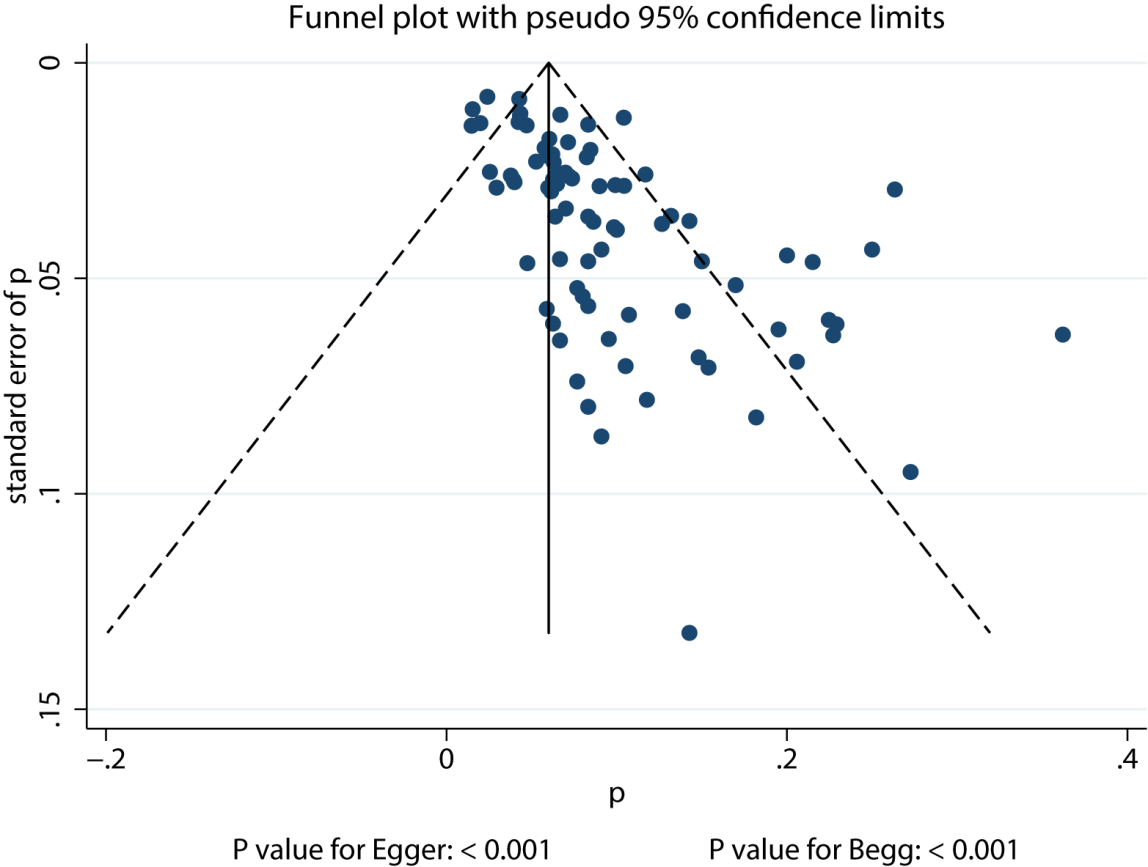
*

*Figure S6. Publication bias test for the pooled incidence of stroke after stenting*

*
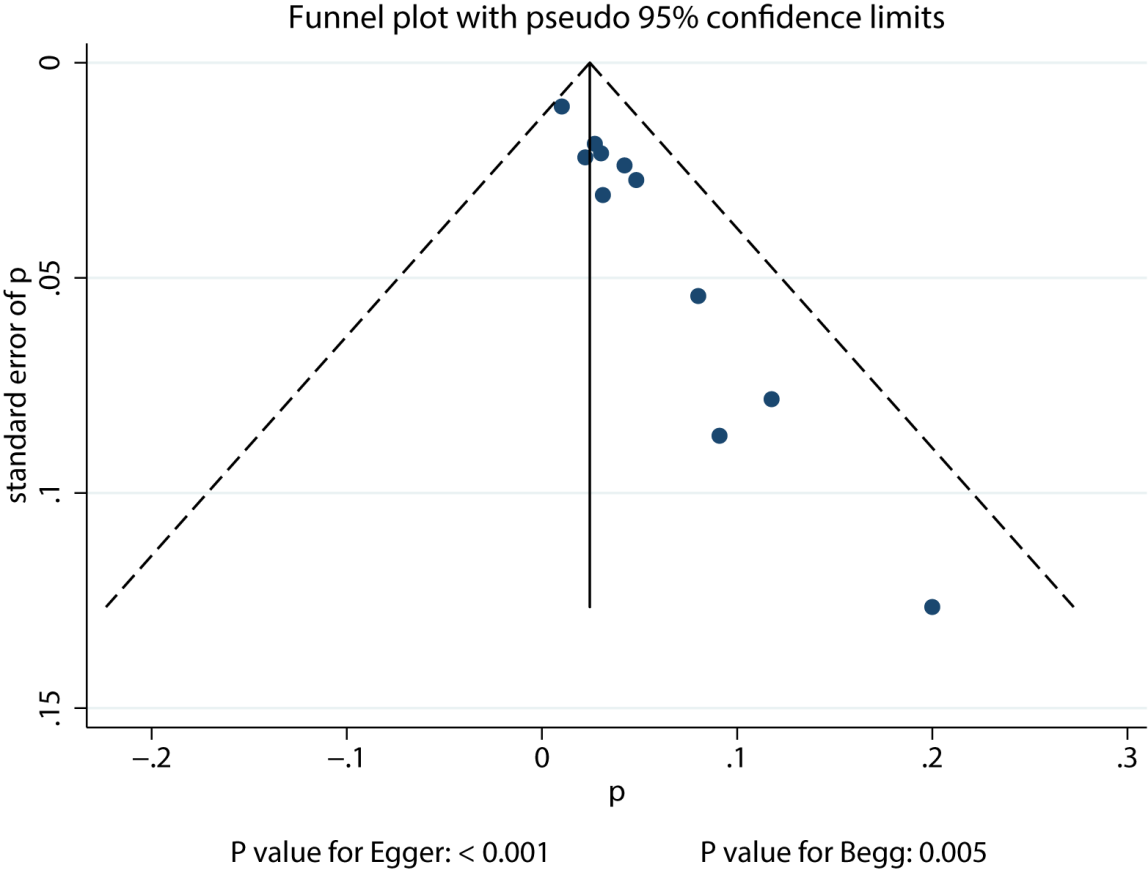
*

*Figure S7. Publication bias test for the pooled incidence of death after balloon angioplasty*

*
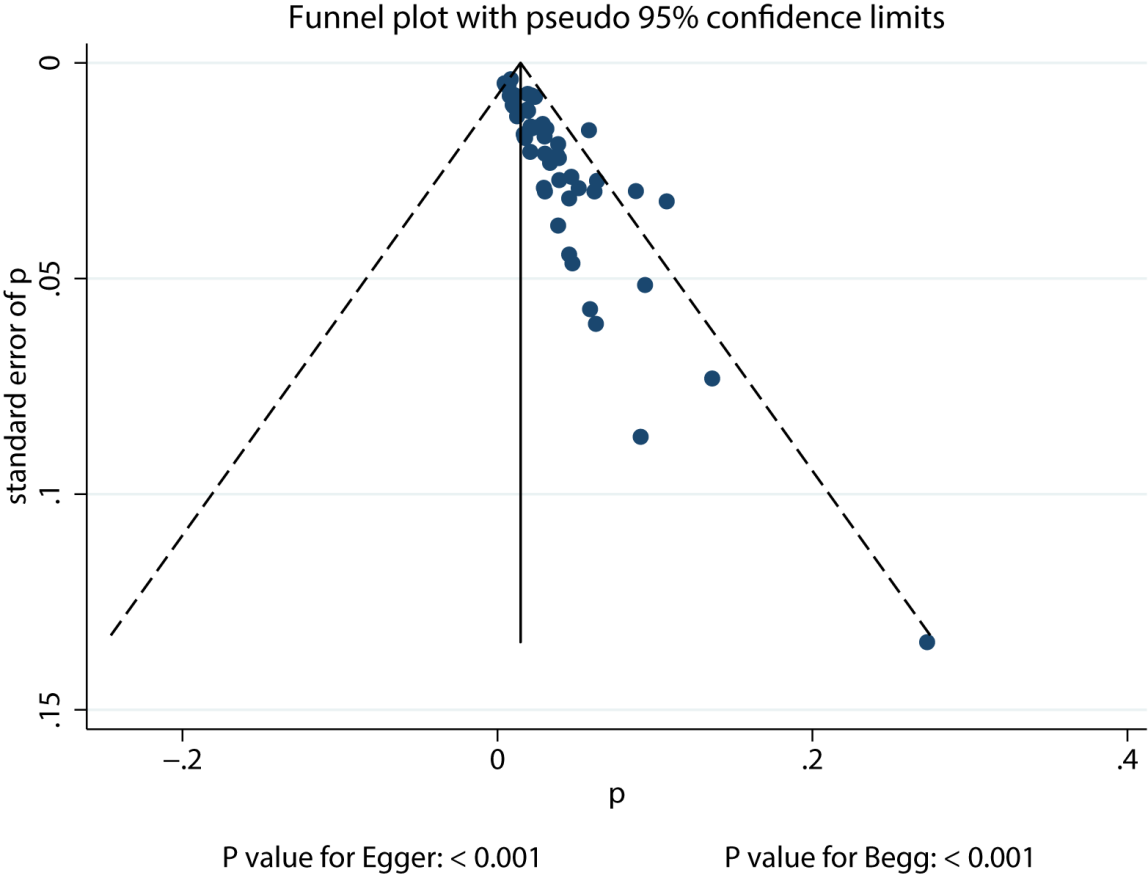
*

*Figure S8. Publication bias test for the pooled incidence of death after stenting*

*
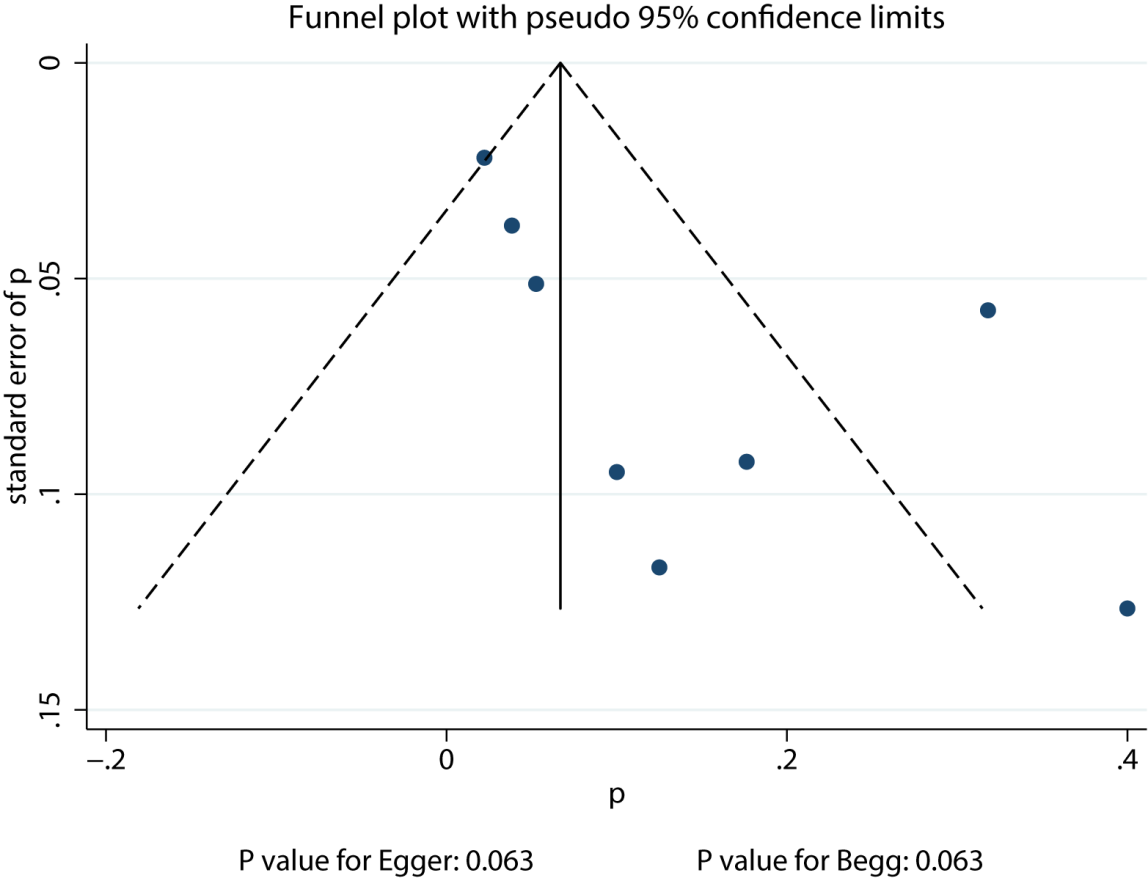
*

*Figure S9. Publication bias test for the pooled incidence of dissection after balloon angioplasty*

*
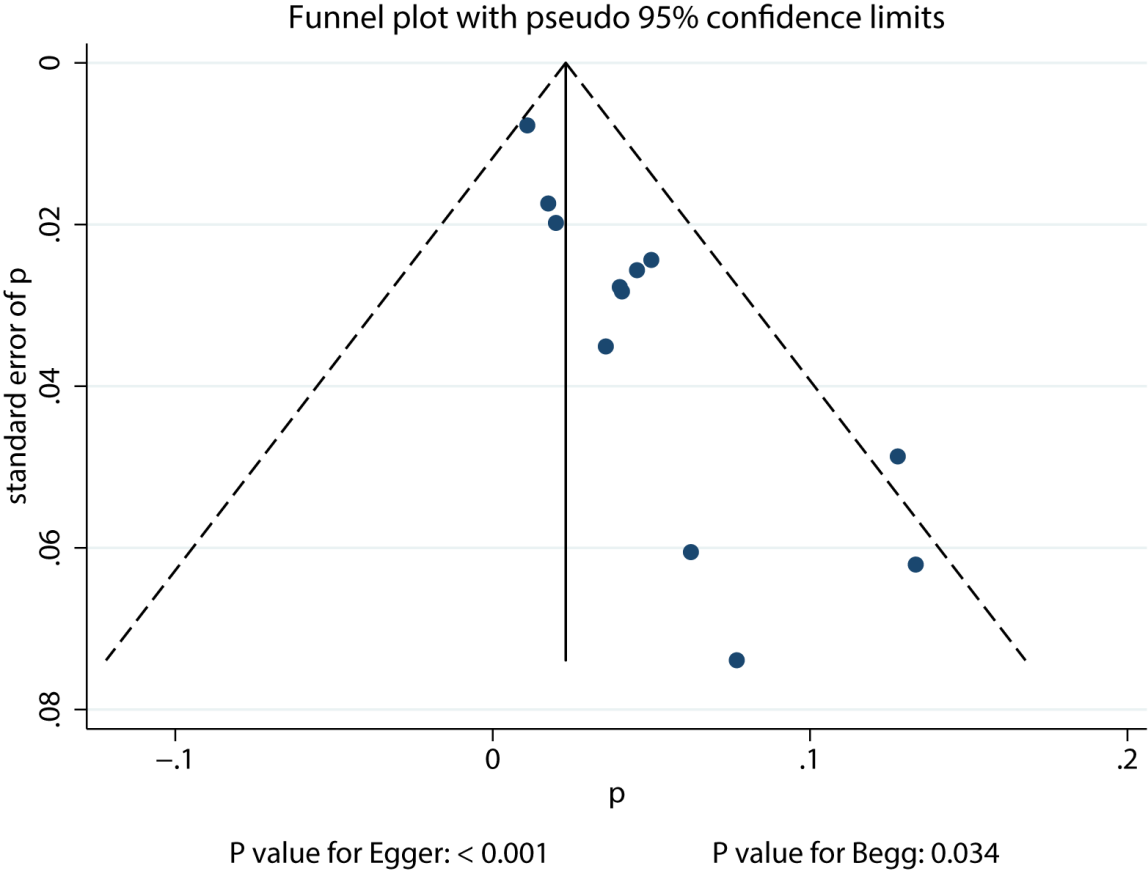
*

*Figure S10. Publication bias test for the pooled incidence of dissection after stenting*
